# Alveolar epithelial type 2 cell specific loss of IGFBP2 activates inflammation in COVID-19

**DOI:** 10.1186/s12931-025-03187-9

**Published:** 2025-03-22

**Authors:** Valentina Pujadas, Chiahsuan Chin, Narendra V. Sankpal, James Buhrmaster, Ashwini Arjuna, Rajat Walia, Michael A. Smith, Oliver Eickelberg, Ross M. Bremner, Thalachallour Mohanakumar, Angara Sureshbabu

**Affiliations:** 1 Norton Thoracic Institute, St. Joseph’s Hospital and Medical Center, 124 W. Thomas Road, Ste. 100, Phoenix, AZ 85013 USA; 2 Creighton University School of Medicine — Phoenix Regional Campus, Phoenix, AZ USA; 3 Division of Pulmonary, Allergy, Critical Care and Sleep Medicine, University of Pittsburgh Medical Center, Pittsburgh, PA USA

## Abstract

The coronavirus disease 2019 (COVID-19) global pandemic is caused by severe acute respiratory syndrome coronavirus 2 (SARS-CoV-2). However, our understanding of SARS-CoV-2-induced inflammation in alveolar epithelial cells remains very limited. The contributions of intracellular insulin-like growth factor binding protein-2 (IGFBP2) to SARS-CoV-2 pathogenesis are also unclear. In this study, we have uncovered a critical role for IGFBP2, specifically in alveolar epithelial type 2 cells (AEC2), in the immunopathogenesis of COVID-19. Using bulk RNA sequencing, we show that *IGFBP2* mRNA expression is significantly downregulated in primary AEC2 cells isolated from fibrotic lung regions from patients with COVID-19-acute respiratory distress syndrome (ARDS) compared to those with idiopathic pulmonary fibrosis (IPF) alone or IPF with a history of COVID-19. Using multicolor immunohistochemistry, we demonstrated that IGFBP2 and its selective ligands IGF1 and IGF2 were significantly reduced in AEC2 cells from patients with COVID-ARDS, IPF alone, or IPF with COVID history than in those from age-matched donor controls. Further, we demonstrated that lentiviral expression of *Igfbp2* significantly reduced mRNA expression of proinflammatory cytokines—Tnf-α, Il1β, Il6, Stat3, Stat6 and chemokine receptors—Ccr2 and Ccr5—in mouse lung epithelial cells challenged with SARS-CoV-2 spike protein injury (S2; 500 ng/mL). Finally, we demonstrated higher levels of cytokines—TNF-α; IL-6 and chemokine receptor—CCR5 in AEC2 cells from COVID-ARDS patients compared to the IPF alone and the IPF with COVID history patients. Altogether, these data suggest that anti-inflammatory properties of IGFBP2 in AEC2 cells and its localized delivery may serve as potential therapeutic strategy for patients with COVID-19.

**Graphical Abstract:**

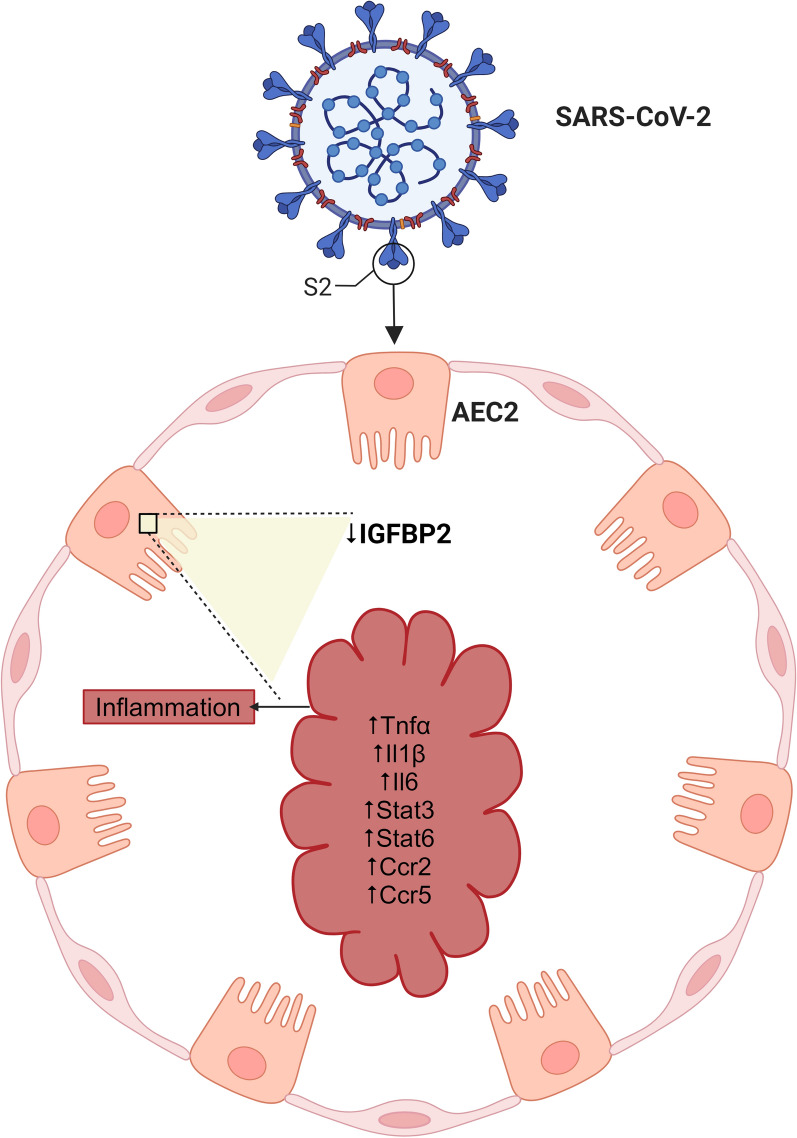

**Supplementary Information:**

The online version contains supplementary material available at 10.1186/s12931-025-03187-9.

## Introduction

Severe acute respiratory syndrome coronavirus 2 (SARS-CoV-2) is the cause of the recent coronavirus disease 2019 (COVID-19) pandemic. After entering through the nasal cavity, SARS-CoV-2 primarily infects ciliated cells and is released into the respiratory tracts [1]. As the virus spreads, the clinical manifestation varies from mild symptoms to severe respiratory distress syndrome or death [2]. Through the interaction of the spike glycoprotein with the angiotensin converting enzyme (ACE2) receptor, SARS-CoV-2 infects human cells including alveolar epithelial cells [3]. The extension of the SARS-CoV-2 infection into the alveolar space produces progressive hypoxia associated with pulmonary infiltration. Further, SARS-CoV-2 infection of alveolar cells induces alveolar flooding and inflammatory cell infiltration [4]. Pathological processes include diffuse alveolar damage, hyaline membrane formation and epithelial and microvascular injury [5, 6]. Nevertheless, pulmonary fibrosis is the most significant long-term complication of COVID-19 [7, 8].

In the lungs, an equilibrium of proinflammatory and anti-inflammatory responses are vital for immune homeostasis. However, a cytokine storm triggered by SARS-CoV-2 infection can result in a severe clinical manifestation known as COVID-acute respiratory distress syndrome (COVID-ARDS) [9, 10]. During the pathogenesis of COVID-ARDS, elevated levels of cytokines and chemokines are released by both alveolar macrophages and epithelial cells [11]. Single-cell RNA sequencing studies have shown that alveolar type II epithelial cells (AEC2) are susceptible to SARS-CoV-2 infection enabled by ACE2 receptors and TMPRSS2 protease activity [12, 13]. Mostly, the infection of AEC2 cells by SARS-CoV-2 that drives ARDS in severe cases of COVID-19 could be a major contributor to ARDS-induced fibrosis. Furthermore, a model system of induced pluripotent stem cell-derived AEC2 cells after COVID-infection revealed increased expression of proinflammatory cytokine genes including *IL-6, TNF, CXCL-12,* and *CXCL8* and the downregulation of surfactant-associated genes including *SFTPC, SFTPA1,* and *LAMP3* [14]. Transcriptome analysis of a 3D organoid AEC2 cell culture revealed differential expression of interferon-associated genes, and immunological analyses showed loss of surfactant protein C and caspase 3, recapitulating features of COVID-19 lungs [15]. Inflammatory cytokines are released by infection of the alveolar epithelial cells, and consequently may trigger a profibrotic macrophage response and lung fibrosis through the accumulation of macrophages [16]. Because AEC2 cells act like tissue stem cells in the alveolar space, their dysfunction not only attracts more macrophages but also diminishes regenerative ability, thus aggravating pulmonary fibrosis. Therefore, it is crucial to understand how SARS-CoV-2 infection changes the differential expression and signaling pathways of AEC2 cells. These insights pave the development of new anti-inflammatory therapies that are needed to reduce secondary impacts of SARS-CoV-2 infection.

Insulin-like growth factor binding protein 2 (IGFBP2) plays pivotal roles in cellular processes including but not limited to proliferation, differentiation, and senescence [17]. Our previous study demonstrated that loss of *IGFBP2* function specifically in AEC2 cells promotes lung fibrosis [18]. In the present study, using total RNA sequencing, we aimed to discover the potential mechanisms that damage the injured AEC2 cells after SARS-CoV-2 infection. Among the identified differential genes, we focused on *IGFBP2*, which could regulate local inflammatory conditions in AEC2 cells. Therefore, we investigated whether IGFBP2 regulates inflammatory cytokines and chemokines specifically in AEC2 cells following SARS-CoV-2 spike protein injury. Using bulk RNA sequencing and multicolor-immunohistochemistry, we demonstrated that IGFBP2 was significantly reduced in AEC2 cells from patients with COVID-ARDS, IPF alone, or IPF with a history of COVID-19 (IPF with COVID history) compared with healthy donor controls. Using lentiviral expression approaches, we show that IGFBP2 significantly reduced mRNA expression of pro-inflammatory cytokines—Tnf-α, Il1β, Il6, Stat3, Stat6—and chemokine receptors—Ccr2 and Ccr5 —in mouse lung epithelial cells after exposure to the SARS-CoV-2 spike (S2) protein. We also demonstrate that lentiviral transduction of IGF1 significantly increased mRNA expression of cytokines—Tnf-α, Il1β, Il6 and chemokine receptors—Ccr2, Ccr5 and Irf3; whereas lentiviral transduction of IGF2 significantly increased Tnf-α and Ccr5 mRNA expression in mouse lung epithelial cells following SARS-CoV-2 spike protein exposure. In addition, we showed that cytokines, TNF-α and IL-6 and chemokine, CCR5 protein levels were significantly elevated in AEC2 cells from patients with COVID-ARDS compared with those from patients with IPF alone or IPF with COVID history. Collectively, these findings suggest that *IGFBP2* is downregulated in AEC2 cells and its targeted expression may regulate SARS-CoV-2-induced inflammation, and therefore yield therapeutic options for patients with COVID-19.

## Results

### Human lung histology of severe and moderate COVID-19

**Fig. 1:**
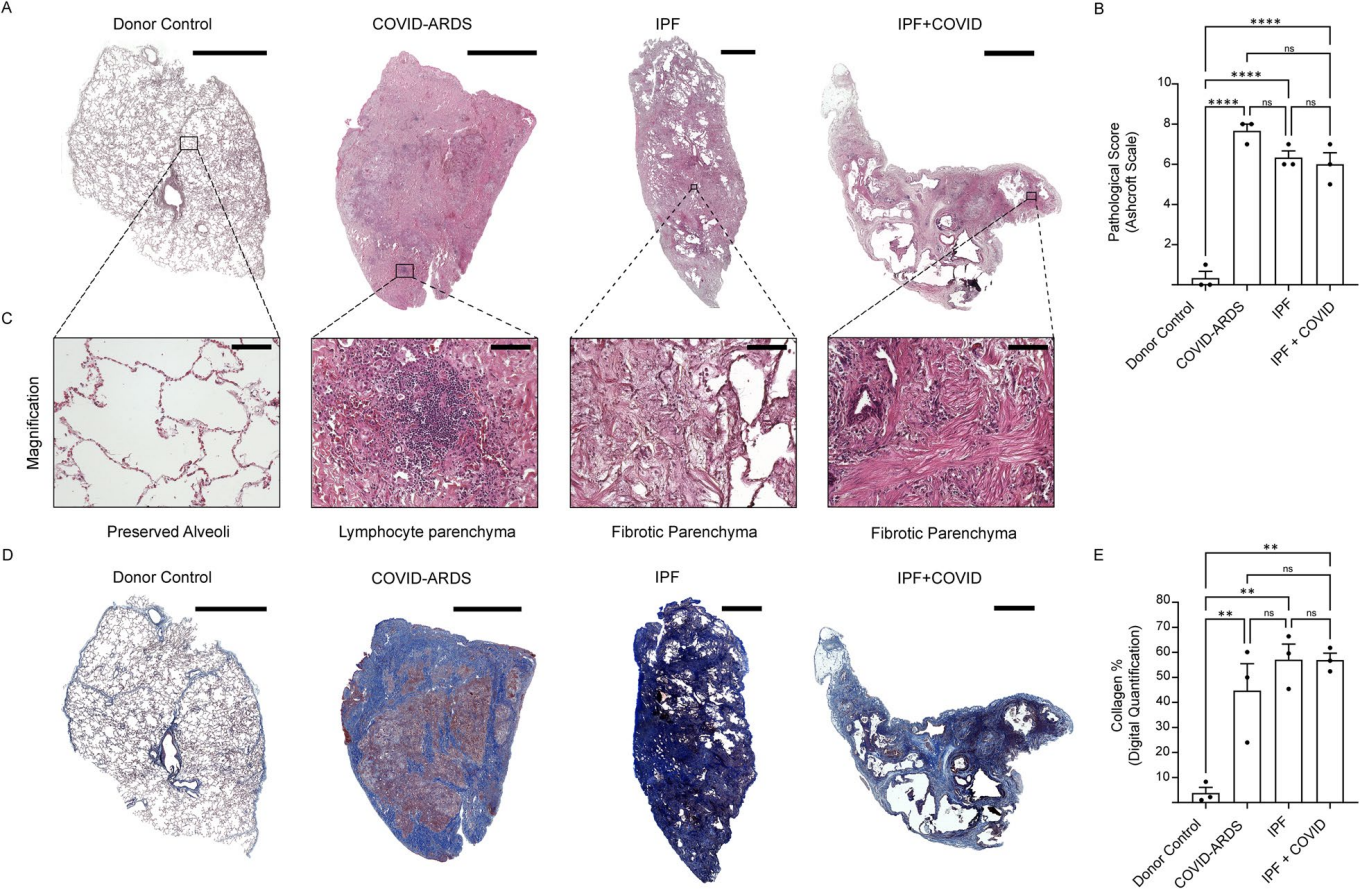
Pathology of human lung sections from patients with COVID-acute respiratory distress syndrome (ARDS), idiopathic pulmonary fibrosis (IPF), or IPF with a history of COVID-19. Representative images of (**A**) H&E and (**D**) Masson’s trichrome stained fibrotic lung sections of donor (healthy) controls and patients with COVID-ARDS, IPF alone, or IPF with COVID history. Scale bars, 3000 μm. **B** Quantitation of pathological score for the H&E-stained lung images from patients with COVID-ARDS, IPF alone, or IPF with COVID history. **C** Bottom panels are showing magnified H&E histological staining images from donor (healthy) controls and patients with COVID-ARDS, IPF alone, or IPF with COVID history. Scale bars, 100 μm. **E** Digital quantification of Masson’s trichrome-stained lung images from patients with COVID-ARDS, IPF alone, or IPF with COVID history. Data are presented as mean ± SEM. NS, not significant; ****p < 0.0001, ANOVA. N = 3 for each group (Donor (healthy) controls, COVID-ARDS, IPF alone, and IPF with COVID history)

To examine the histological changes driven by SARS-CoV-2 infection, we performed H&E and Masson’s trichrome staining in lung regions obtained from patients diagnosed with COVID-ARDS (severe), IPF alone, or IPF with COVID history (moderate). H&E staining analyses revealed abnormal lung structures with heterogeneity in COVID-ARDS patients compared to patients from the other disease groups. Ashcroft’s scale analyses showed a significantly higher pathological score in all lung disease groups compared to donor controls (Fig. 1A, B). In addition, lymphocytic parenchymal infiltration was observed in lung regions of patients diagnosed with COVID-ARDS compared to fibrotic parenchyma seen in patients diagnosed with IPF alone, or IPF with COVID history (Fig. 1C). Next, we performed Masson’s trichrome staining to evaluate fibrosis content. Trichrome staining revealed significantly increased collagen content in lungs from all disease groups compared with donor controls (Fig. 1D, E). Together, these data suggest that total lung collagen content was significantly higher in patients with both COVID and non-COVID lung diseases.

### Bulk RNA-sequencing analyses of AEC2 cells in COVID-ARDS and IPF with COVID history

**Fig. 2:**
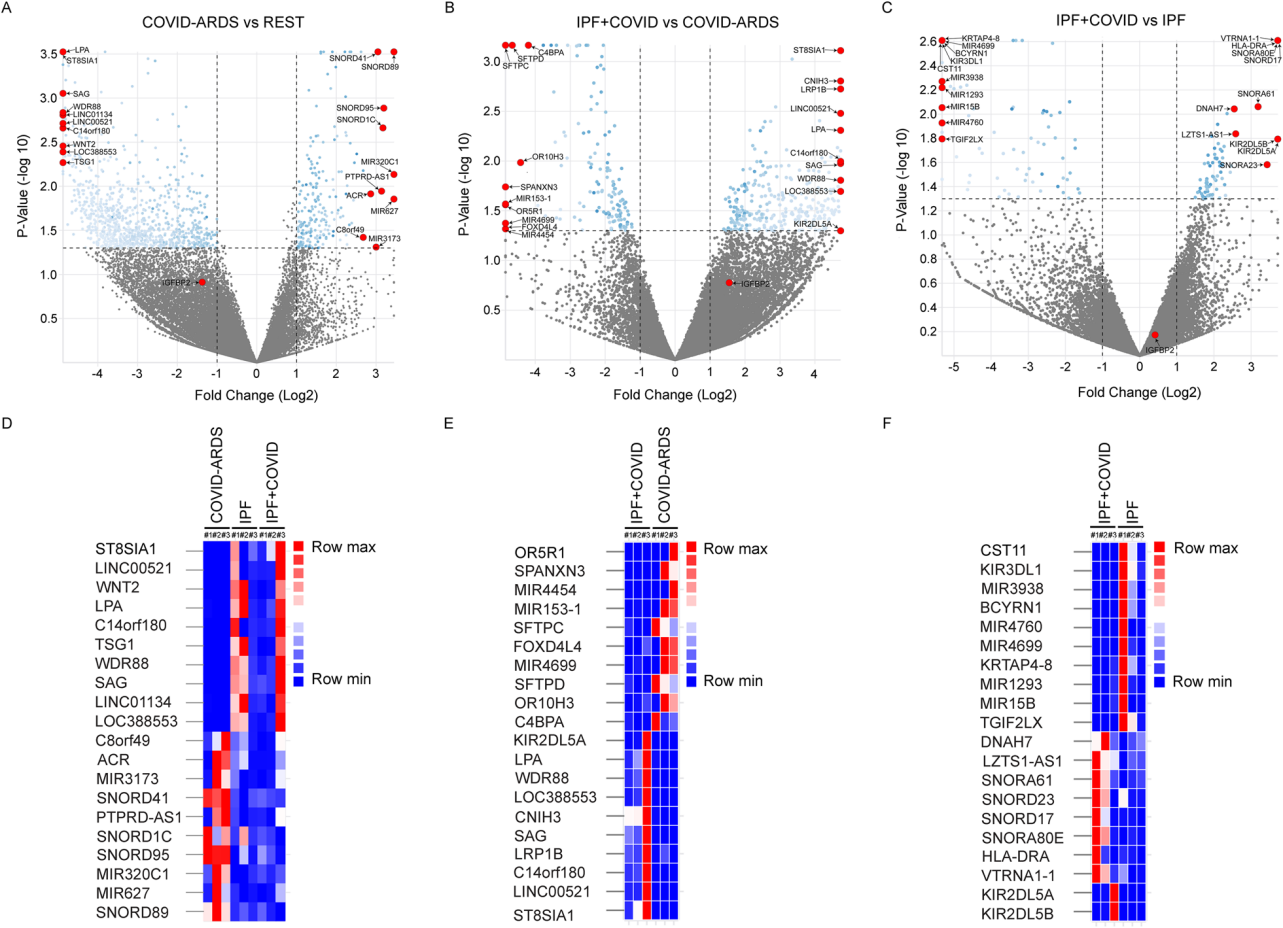
Bulk RNA-seq reveals differential gene expression profile in AEC2 cells. Bulk RNA sequencing was performed to detect differential gene expression in isolated primary AEC2 cells obtained from patients with COVID-acute respiratory distress syndrome (ARDS), idiopathic pulmonary fibrosis (IPF), or IPF with a history of COVID-19. Top panel: Volcano plots showing transcripts that are differentially expressed in (**A**) COVID-ARDS group compared to IPF alone and IPF with COVID history groups combined. **B** IPF with COVID history group compared to COVID-ARDS group (**C**) IPF with COVID history group compared to IPF alone group. Bottom panel: Heat maps showing top 10 differentially expressed genes in (**D**) COVID-ARDS group compared to IPF alone and IPF with COVID history groups combined (**E**) IPF with COVID history group compared to COVID-ARDS group (**F**) IPF with COVID history group compared to IPF alone group. *p ≤ 0.05; N = 3 for each group (COVID-ARDS, IPF with COVID history and IPF alone)

To further investigate the transcriptomic changes between patients with COVID-ARDS, IPF alone, or IPF with moderate COVID history, we performed bulk RNA sequencing in primary AEC2 cells isolated from fibrotic lung regions of the respective patients. Differential gene expression analysis identified top upregulated genes, namely *SNORD89, MIR627, MIR320C1, SNORD95, SNORD1C, PTPRD-AS1, SNORD41, MIR3173, ACR, C8ORF49*, and top downregulated genes, namely *ST8SIA1, LINC00521, WNT2, LPA, C14orf180, TSG1, WDR88, SAG, LINC01134, LOC388553* in the COVID-ARDS group compared to the other lung disease groups combined (IPF alone and IPF with COVID history). Similarly, differential gene expression analysis identified top upregulated genes, namely *ST8SIA1, LINC00521, C14orf180, LRP1B, SAG, CNIH3, LOC388553, WDR88, LPA*, *KIR2DL5A* and top downregulated genes, namely *OR5R1, SPANXN3, MIR4454, MIR153-1, SFTPC, FOXD4L4, MIR4699, SFTPD, OR10H3, C4BPA* in the IPF with COVID history group compared to the COVID-ARDS group. Whereas, differential gene expression analysis identified top upregulated genes, namely *KIR2DL5A, KIR2DL5B, VTRNA1-1, HLA-DRA, SNORA80E, SNORD17, SNORA23, SNORA61, LZTS1-AS1, DNAH7* and top downregulated genes, namely *CST11, KIR3DL1, MIR3938, BCYRN1, MIR4760, MIR4699, KRTAP4-8, MIR1293, MIR15B, TGIF2LX* in the IPF with COVID history group compared to IPF alone group (Fig. 2A, C; S1; Tables S2 – S5).

Recently, IGFBP2 was demonstrated to play a critical role specifically in AEC2 cells in IPF [18]. Therefore, we further examined *IGFBP2* mRNA expression in the aforementioned lung disease groups. The RNA-seq analyses revealed that *IGFBP2* was significantly downregulated in AEC2 cells from the COVID-ARDS group compared with those from other lung disease groups combined (IPF alone and IPF with COVID history) (Fig. 2A, C). Overall, these results indicate that *IGFBP2* transcript levels were low in AEC2 cells of both severe and moderate-COVID mediated lung fibrosis.

### IGFBP2 downregulation in AEC2 cells of COVID-ARDS and IPF with COVID history

**Fig. 3:**
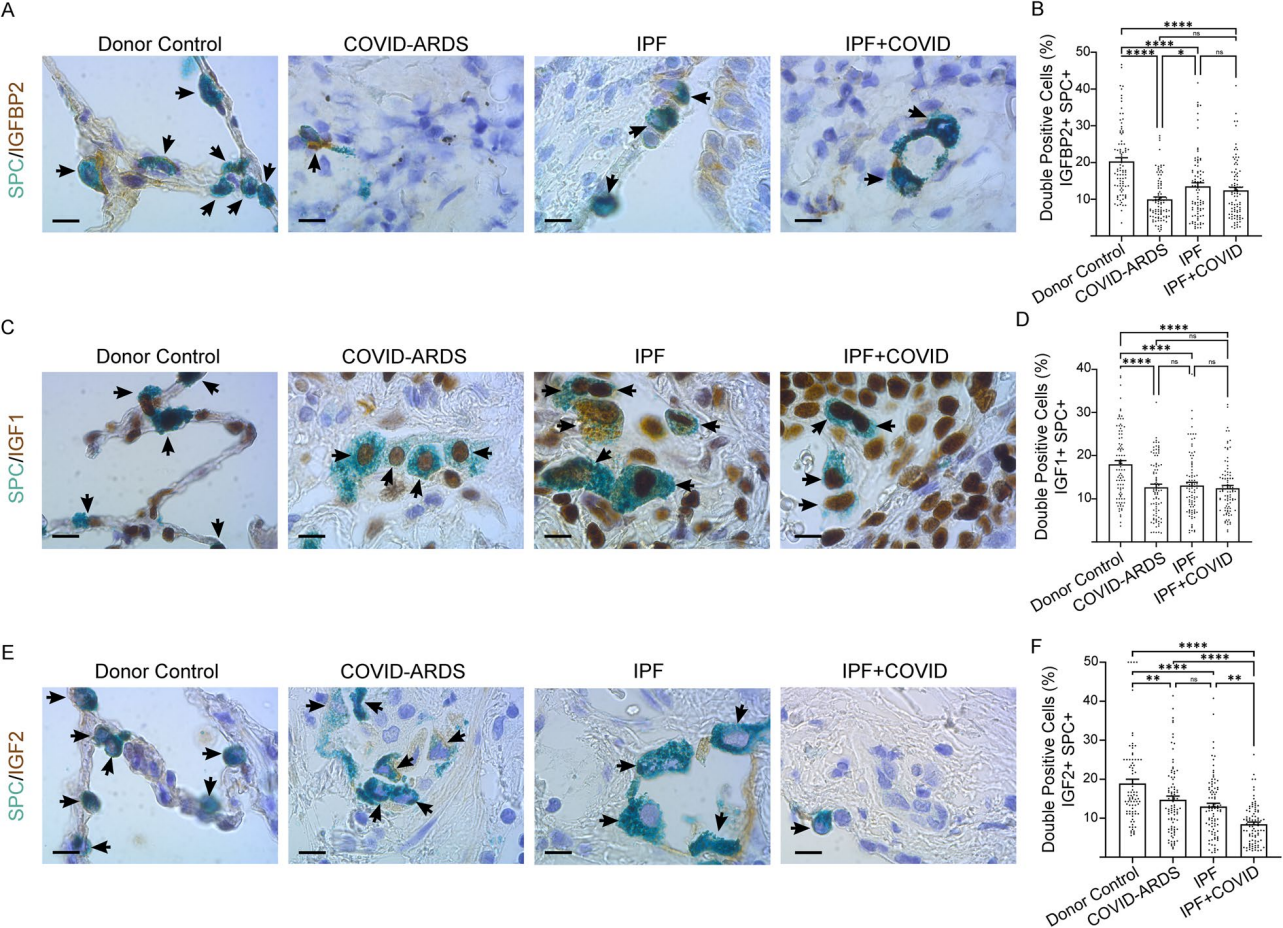
Reduced IGFBP2 protein expression in AEC2 cells of both COVID-infected lungs and lungs without COVID. Multicolor immunohistochemistry images showing fibrotic lung sections stained with SPC (green) and (**A**) IGFBP2; **C** IGF1; and **E** IGF2 (brown) from donor (healthy) controls and patients with COVID-ARDS, idiopathic pulmonary fibrosis (IPF), or IPF with a history of COVID. 25 – 30 images were analyzed per human subject. Scale bars, 10 μm. Quantification of double positive cells for (B) IGFBP2; **D** IGF1; and (**F**) IGF2 in SPC + cells in the fibrotic lung regions of patients with COVID-ARDS, IPF alone, or IPF with COVID history and healthy (donor) controls. Data are presented as mean ± SEM. NS, not significant; *p < 0.05, **p < 0.01, and ****p < 0.0001, One-way ANOVA followed by Tukey post-hoc test. N = 3 for each group (Donor controls, COVID-ARDS, IPF alone, and IPF with COVID history)

Next, we examined the IGFBP2 protein expression levels in lung regions obtained from patients diagnosed with COVID-ARDS, IPF alone, or IPF with COVID history. Using multicolor immunohistochemistry, we demonstrated that protein expression levels of IGFBP2 as well as its selective ligands IGF1 and IGF2 were significantly downregulated in AEC2 cells of the lung disease groups compared with healthy controls. Interestingly, protein expression levels of IGFBP2 in AEC2 cells were significantly lower in the COVID-ARDS group compared with the IPF alone, or IPF with COVID history disease groups. Furthermore, levels of IGF2 protein expression in AEC2 cells were significantly lower in the IPF with COVID history group compared with IPF alone or COVID-ARDS disease groups (Fig. 3A, F). Together, these results indicate reduced expression of IGFBP2 and its selective ligands IGF1 and IGF2 specifically in AEC2 cells from patients diagnosed with severe or moderate COVID-19.

### Lentiviral expression of Igfbp2 reduces inflammation after SARS-CoV-2 spike protein injury

The SARS-CoV-2 spike glycoprotein is a strong inflammatory stimulus that induces the release of proinflammatory cytokines and chemokines [19, 20] and is also the primary target of virus-neutralizing antibodies [21]. The SARS-CoV-2 virus contains a crucial spike(S) protein that consists of two distinct components—S1 and S2 subunits. The S1 RBD domain is part of a highly mutable region and is not an ideal target site for a specific drug. Whereas, the HR (heptad repeat) region within the S2 subunit is crucial for human coronavirus infections, with interaction patterns between HR1 and HR2 [22]. Therefore, we sought to determine whether IGFBP2 plays a role in SARS-CoV-2 spike (S2) glycoprotein-induced inflammatory responses. First, we engineered mouse lung epithelial cells (MLE-12) with a lentivirus vector encoding constitutively expressed proteins—IGFBP2 and its ligands, IGF1 and IGF2. To this end, we measured mRNA expression of cytokines and chemokines in MLE-12 cells challenged with SARS-CoV-2 spike protein injury.

**Fig. 4:**
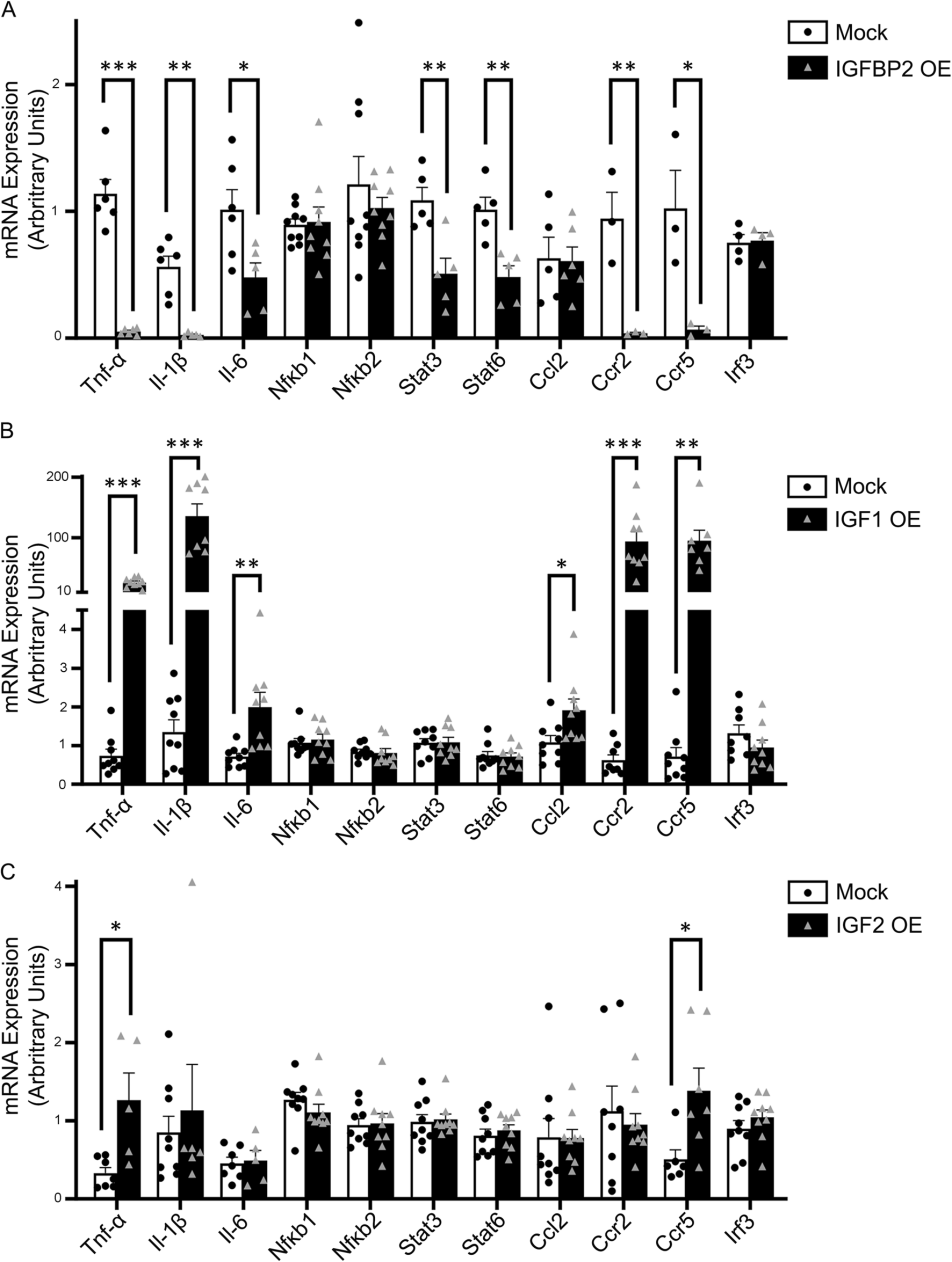
Lentiviral *Igfbp2* expression inhibits COVID S2 spike glycoprotein-induced inflammatory phenotype in MLE-12 cells. Quantitative polymerase chain reaction (QPCR analyses of mRNA expression of a panel of cytokines and chemokines in lentiviral mediated transduction of MLE-12 cells in the absence or presence of SARS-COV-2 spike protein S2 treatment. Relative mRNA expression of cytokines and chemokines in MLE-12 cells expressing (**A**) IGFBP2 versus mock (**B**) IGF1 versus mock (**C**) IGF2 versus mock after S2 spike protein (500 ng/ml) injury at 24 h. Data are representative of minimum of 3 independent experiments. Data are presented as mean ± SEM. *p < 0.05, **p < 0.01, and ***p < 0.001, Student’s unpaired two-tailed t test

Quantitative RT-PCR analysis revealed that lentivirally expressed IGFBP2 significantly decreased mRNA expression of proinflammatory cytokines—Tnf-α, Il1β, Il6, Stat3 and Stat6—and chemokines—Ccr2 and Ccr5—compared with mock transfection of MLE-12 cells after SARS-CoV-2 spike protein challenge (Fig. 4A; S2). Further, we examined the role of selective ligands—IGF1 and IGF2—in SARS-CoV-2-induced inflammatory responses in MLE-12 cells. Intriguingly, lentivirus transduced IGF1 significantly increased mRNA expression of proinflammatory cytokines—Tnf-α, Il1β, Il6—and chemokines—Ccl2, Ccr2, and Ccr5, whereas, IGF2 overexpression significantly increased Tnf-α and Ccr5 mRNA expression compared to mock-transfection of MLE-12 cells after SARS-CoV-2 spike protein stimuli (Fig. 4B, C; S2). Overall, these data indicate that IGFBP2 regulates inflammatory cytokines and chemokines in MLE-12 cells following exposure to SARS-CoV-2 spike protein stimuli.

### Inflammatory cytokine and chemokine expression in alveolar epithelial cells of severe and moderate COVID-19

**Fig. 5:**
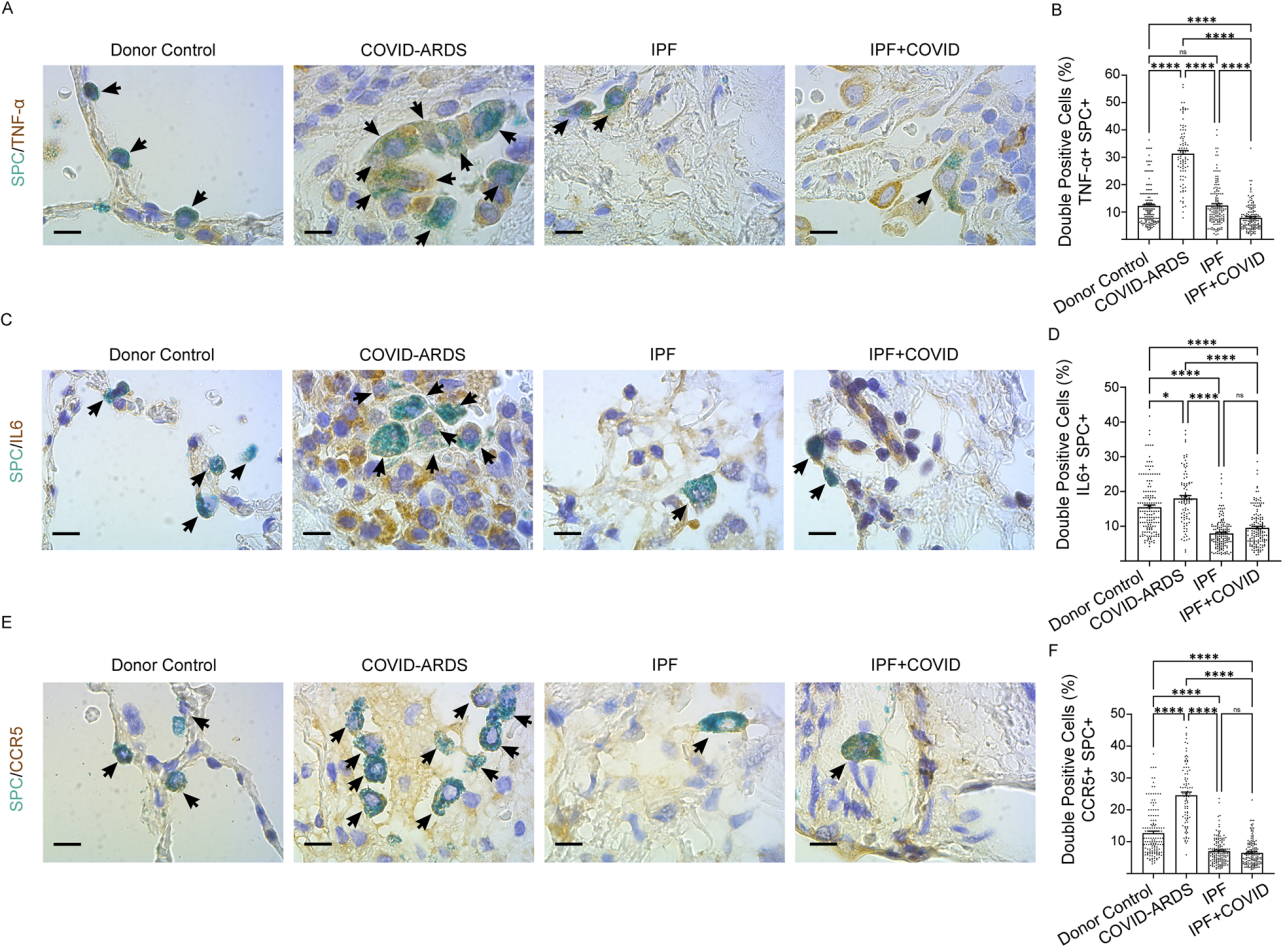
Higher TNF-α; IL6 and CCR5 protein expression in AEC2 cells of severe COVID-infected lungs. Multicolor immunohistochemistry images showing fibrotic lung sections stained with SPC (green) and (**A**) TNF-α (**C**) IL-6 (brown) (**E**) CCR5 from donor (healthy) controls and patients with COVID-ARDS, IPF alone, or IPF with COVID history. 25—30 images were analyzed per human subject. Scale bars, 10 μm. Quantification of double positive cells for (**B**) TNF-α (D) IL-6 (**F**) CCR5 from donor (healthy) controls and patients with COVID-ARDS, IPF alone, or IPF with COVID history. Data are presented as mean ± SEM. *p < 0.05, **p < 0.01, and ****p < 0.0001, One-way ANOVA followed by Tukey post-hoc test. N = 3 for COVID-ARDS group, N = 5 for each group (Donor Control; IPF alone, and IPF with COVID history)

To further investigate the role of COVID-induced inflammation, specifically in AEC2 cells, we performed multicolor immunohistochemical analyses of cytokine and chemokine expression in fibrotic lung regions of patients diagnosed with COVID-ARDS, IPF alone, or IPF with COVID history. —TNF-α and IL-6—play significant roles in several inflammatory diseases including COVID-19 [23, 24]. Chemokine receptor, CCR5 expression was elevated in critically ill patients with severe COVID-19 [25]. Multicolor immunohistochemical analyses revealed TNF-α protein expression levels in AEC2 cells were significantly increased in patients with COVID-ARDS compared to donor controls and those with IPF alone or IPF with COVID history. Consistent with this, IL-6 protein expression levels in AEC2 cells were significantly higher in patients with COVID-ARDS compared to those with IPF alone or IPF with COVID history (Fig. 5A, D). Surprisingly, an increase in IL-6 in the lungs of donor controls was found as compared to those with IPF alone or IPF with COVID history (Fig. 5C, D). This may have been affected by comorbidities, frailty and systemic involvement of elderly individuals [26, 27]. Similarly, CCR5 protein expression levels were significantly increased in AEC2 cells from patients with COVID-ARDS compared to donor controls and those with IPF alone or IPF with COVID history (Fig. 5E, F). Overall, these findings suggest that cytokines — TNF-α and IL-6, and chemokine receptor CCR5 were significantly elevated in AEC2 cells from patients with COVID-ARDS as compared to those from patients with IPF alone or IPF with COVID history.

## Discussion

The COVID-19 pandemic has caused more than 704 million infections and 7 million deaths worldwide [28]. Prolonged levels of inflammatory responses are commonly observed in patients with COVID-19, thus, elucidating the underlying mechanisms will pave the way for the development of novel therapeutic targets [29]. In the present study, we demonstrate that IGFBP2 is involved in the negative regulation of proinflammatory responses after SARS-CoV-2 spike protein injury. Consistently, we observed that the protein expression levels of IGFBP2 and its selective ligands IGF1 and IGF2 were significantly lower in AEC2 cells from patients with severe or moderate SARS-CoV-2 infection compared to those from donor (healthy) controls. Furthermore, we found that *Igfbp2* expression downregulated proinflammatory cytokines—Tnf-α, Il1β, Il6, Stat3, Stat6—and chemokines—Ccr2, Ccr5 —in mouse lung epithelial cells after SARS-CoV-2 spike protein injury. We also showed that TNF-α, IL-6 and CCR-5 levels of protein expression were higher in AEC2 cells from patients with COVID-ARDS compared to those from patients with IPF alone or IPF with COVID history. Collectively, our findings demonstrate a potential role for IGFBP2 in suppressing the proinflammatory responses specifically in local AEC2 cells during SARS-CoV-2 infection.

Recent studies have demonstrated that viral infection can progress to ARDS and induce long-term complications such as lung fibrosis [30–32]. Thus, in the current study we assessed the impact of lung fibrosis on modulation of SARS-CoV-2 infection. First, we assessed disease severity in the lung tissues obtained from patients with severe to moderate SARS-CoV-2 infection. We found that the disease score was significantly higher in lung regions from patients with severe or moderate SARS-CoV-2 infection compared to those from healthy controls. Similarly, trichrome staining showed significantly increased collagen content in both severe and moderate SARS-CoV-2 infected lung regions compared with healthy controls. These findings suggest that SARS-CoV-2 infection may provide distinct signals at early time points that are unique to end-stage pulmonary fibrosis and understanding these responses is critical for the development of targeted therapies for COVID-19.

A previous study by our group demonstrated that downregulation of IGFBP2 mediates senescence specifically in AEC2 cells through the P21 signaling pathway, contributing to the development of pulmonary fibrosis. Furthermore, significantly reduced expression of IGFBP2 was evident in AEC2 cells of patients with IPF and/or IPF with pulmonary arterial hypertension [18]. Previous studies have shown that other IGFBPs regulate inflammatory responses in both physiological and pathological settings [33–35]. In the current study, we demonstrated that reduced IGFBP2 expression represents a novel pathogenic mechanism for alveolar epithelial cell inflammation in the pathogenesis of COVID-19. In addition, we found a negative association between levels of IGFBP2 and levels of TNF-α, IL-6 and CCR-5 specifically in AEC2 cells from patients with COVID-ARDS relative to those from patients with IPF alone or IPF with COVID history.

Excessive SARS-CoV-2-associated inflammation contributes to morbidity and mortality. SARS-CoV-2 infection is driven by differential induction of pro- and anti- inflammatory responses [36, 37]. Moreover, cytokine release syndrome characterized by TNF-α, IL-6, and IL-1β was associated with disease severity [38] and, higher levels of IL6 were more predictive for COVID-19 mortality [39, 40]. Our results indicate that IGFBP2 expression dampens expression of central cytokines—Tnf-α, Il6, and Il1β as well as Stat3 and Stat6 in mouse lung epithelial cells after SARS-CoV-2 spike protein injury. Consistent with cytokines, we also demonstrated IGFBP2 decreases expression of chemokines—Ccr2 and Ccr5 in mouse lung epithelial cells following SARS-CoV-2 spike protein injury. Of note, *CCR2* and *CCR5* share sequence homology, presumably resulting from a gene duplication event [41]. Given the role of CCR5 in respiratory virus infections, recent study suggests that CCR5-Δ32 (a CCR5 variant) may be protective against SARS-CoV-2 infection [42]. While CCR2 is mainly considered proinflammatory; it has been shown to exhibit anti-inflammatory functions in certain cell types, such as regulatory T lymphocytes [43]. Of note, imbalance of epigenetic and metabolic regulatory pathways have been implicated in enhanced inflammation [44, 45]. Future studies are required to determine how IGFBP2 inhibits cytokines—TNF-α and IL6—and chemokine—CCR5, are critical for improving COVID-19 outcomes.

This study has some limitations. All patients were from a single-center and had end-stage lung diseases. Given the difficulty of obtaining samples from patients with COVID-ARDS (n = 3), the sample size is small. Additionally, the study did not account for SARS-CoV-2 variant-specific effects on disease outcomes. Furthermore, anti-inflammatory properties of IGFBP2 during other viral infections were not studied.

This study demonstrates that IGFBP2 and its ligands IGF1 and IGF2 are downregulated specifically in AEC2 cells during SARS-CoV-2-infection. Our findings provide evidence that IGFBP2 exert anti-inflammatory properties in mouse lung epithelial cells following SARS-CoV-2 spike protein injury. In addition, loss of IGFBP2 expression is negatively associated with higher expression of cytokines—TNF-α and IL6—and chemokine—CCR5 in AEC2 cells of patients with severe and moderate COVID compared to those with IPF alone. The complex interplay between these inflammatory mediators and the potential regulatory role of IGFBP2 highlights the intricate nature of the immune response to SARS-CoV-2. In conclusion our study reveals that loss of IGFBP2 specifically in AEC2 cells increases inflammation and targeting IGFBP2 could be therapeutically beneficial during COVID-19 infection.

## Methods

### Study participant demographics

All COVID-ARDS patients were diagnosed in accordance with clinical parameters. One COVID-ARDS patient has mild degree of hypoxemia at the time of transplant surgery. All IPF alone patients have no known history of SARS-CoV-2 infection or other viral infection. All moderate COVID-IPF patients have under 6 months of SARS-CoV-2 infection history from the date of transplant surgery. SARS-CoV-2 variants were not considered during this study. The details of patient demographics and clinical characteristics are provided in Table S1.

### Cell culture

The MLE-12 (mouse lung epithelial) cell line was obtained from A.T.C.C. (American Type Culture Collection, Rockville, Maryland, USA), and cultured in DMEM/F12 medium (ScienCell, Carlsbad, CA) supplemented with 10% fetal bovine serum and 50 μg/ml plasmocin (InvivoGen, San Diego, CA) in a humidified atmosphere with 5% CO2 at 37 °C. Since S2 subunit is crucial for human coronavirus infections, recombinant SARS-CoV-2 spike glycoprotein S2 subunit (R&D Systems, Minneapolis, MN; catalog# 10590-CV) was used to treat MLE-12 cells at 500 ng/ml for 24 h.

### Histology preparations

Lung sections of 3–5 μm thickness were cut with a rotary microtome. The sections were stained with hematoxylin (catalog# 1.05174; Millipore Sigma, Burlington, MA) and eosin (Millipore Sigma; Catalog# 1.17081) (H&E) and Masson’s trichrome (Millipore Sigma; catalog# HT-15), according to the manufacturer’s instructions using standard protocols.

### Pathological score and histological digital analyses

The modified Ashcroft score, a pathological scale used to assess the extent of pulmonary fibrosis by examining alveolar septa and overall lung tissue structure, was applied to samples in this study. Digital quantification of the collagen percentages in the Masson's trichrome-stained lung samples was performed using ImageJ 1.54f software (https://imagej.net/). For quantification, whole lung images were acquired by EVOS microscope and subsequent analysis was performed from all analyzed images.

### Isolation of alveolar epithelial type 2 cells

Alveolar epithelial type 2 (AEC2) cells were isolated from human fibrotic lungs obtained from all disease groups as previously described [18]. Briefly, human fibrotic lung tissue was cut into small 1 cm^2^ pieces and enzymatically digested using a mixture of 1 mg/ml of collagenase I and 5 U/ml of Dispase at 37 °C for 25 min. The initial cell separation was performed by using CD45 magnetic beads (catalog # 130-045-801, Miltenyi Biotec) to isolate specific cell populations from the suspension. After filtering the cell mixture through various sized sieving filters (100-, 40-, 20 mm; Pluriselect, USA), suspension cells were treated with DNase and separated into different populations. Following filtration, cells were suspended in MACS buffer and separated by EpCAM magnetic beads (catalog #130-105-958, Miltenyi Biotec). Subsequently, this process resulted in a population of alveolar epithelial type 2 (AEC2) cells, which were then analyzed using RNA sequencing analysis.

### Multicolor immunohistochemistry

Multicolor immunohistochemical staining was performed by the Leica Bond-Rx automated system (Leica Microsystems, Wetzlar, Germany). The green chromogen (Leica Microsystems; catalog# DS9913) was used for prosurfactant protein C (Abcam, Cambridge, United Kingdom; catalog# ab90716, 1:2000), and the bond polymer refine system (Leica Microsystems; catalog# DS9800) was used for the following antibodies IGFBP2 (Abcam; catalog# ab188200, 1:2000), IGF1 (ABclonal, Woburn, MA; catalog# A0830, 1:100), IGF2 (Novus Biologicals; catalog# NBP248510, 1:200), TNF-α (ABclonal; catalog# A11534, 1:5000), IL6 (Bioss Inc; catalog# bs-0782R, 1:150), CCR5 (Proteintech; catalog# 17476–1-AP, 1:800). Images were acquired at 63X magnification by the Zeiss Imager Z1 microscope (Objective Model # 420782–9900).

### RNA sequencing and bioinformatics analyses

For bulk RNA sequencing, total RNA was extracted from primary AEC2 cells isolated from the fibrotic lung regions of patients diagnosed with COVID-ARDS, IPF alone, or IPF with COVID history. The library preparation was performed as per the manufacturer’s instructions (Zymo Research; catalog #R3000). Briefly, RNA Library Prep Kit was used to generate the sequencing library. Total RNA was reverse transcribed and partial adapters P7 and P5 are directly ligated to the cDNA. Libraries were sequenced using an Illumina HiSeq 2500. Genome alignment, STAR analysis and differential expression analysis were performed using workflows on Basepairtech (https://www.basepairtech.com). For the bulk RNA-sequencing analysis, GRCh37/hg19 genome assembly was used. Differential expression analysis between disease groups were performed using DESeq2 with thresholds of |logFC|> 1 and p-value < 0.05. The differentially expressed genes (DEGs) from the disease groups were then subjected to volcano plot and heat mapping analyses, focusing on top 10 genes up and downregulated.

### Lentivirus transduction

Briefly, 1 × 10^6^ HEK293T cells were seeded in a 10-cm dish. The next day, cells were co-transfected with Lipofectamine 3000 and lentiviral plasmids—pΔ8.9, pVSVG and transgene of interest either IGF1 or IGF2. Media were changed the following day. Viral particles were harvested 48 h following the media change. The resulting supernatant was subsequently used for lentiviral transduction of MLE-12 cells. After transduction, MLE-12 cells were stably selected using antibiotic selection conditions. *Igfbp2* lentivirus was commercially obtained (OriGene Technologies; catalog# MR204287L3V) and was used as previously reported [18].

### Quantitative polymerase chain reaction (qPCR)

Total RNA was harvested from 350 × 10^3^ MLE-12 cells using RNeasy^®^ Fibrous Tissue Mini Kit (Qiagen, Valencia, CA; catalog# 74704) according to the manufacturer’s instructions. Concentration of RNA in the sample was measured using a Nanodrop ND-1000 Spectrophotometer (Thermo Fisher, Waltham, MA), and 1 µg was used to synthesize cDNA using Applied Biosciences High-Capacity RNA to cDNA^™^ Kit (Thermo Fisher; catalog# 4387406) according to the manufacturer’s instructions. The following primers were obtained from Realtime Primers (Realtime Primers, LLC) are described in the table below:

**Table:** 

Primers	Source	Sequences
Tnf-α	RealTimePrimers	5'-CCCACTCTGACCCCTTTACT-3' 5'-TTTGAGTCCTTGATGGTGGT-3'
Ccr2	RealTimePrimers	5'-GGAGAAAAGCCAACTCCTTC-3' 5'-AGGCAGTTGCAAAGGTACTG-3'
Ccr5	RealTimePrimers	5'-GGCAACAGAGACTCTTGGAA-3' 5'-TCCTGTGGATCGGGTATAGA-3'
Il-1β	RealTimePrimers	5'-CCCAACTGGTACATCAGCAC-3' 5'-TCTGCTCATTCACGAAAAGG-3'
Il-6	RealTimePrimers	5'-CTACCCCAATTTCCAATGCT-3' 5'-ACCACAGTGAGGAATGTCCA-3'
Irf3	RealTimePrimers	5'-GGAAATATCTGAGCCCCACT -3' 5'-CAGCTCTGGACCTGTCTTGT-3'
Nfκb1 (p105)	RealTimePrimers	5'-TGAGAATGGACAGAACAGCA-3' 5'-AAGCTGAACAAACACGGAAG-3'
Nfκb2 (p49/p100)	RealTimePrimers	5'-ACCTTTGCTGGAAACACACC-3' 5'-GTATCCCTCTCAGGCCCTTC-3'
Stat3	RealTimePrimers	5'-CAATACCATTGACCTGCCGAT-3' 5'-GAGCGACTCAAACTGCCCT-3'
Stat6	RealTimePrimers	5'-CTCTGTGGGGCCTAATTTCCA-3' 5'-CATCTGAACCGACCAGGAACT-3'
Ccl2/Mcp1	RealTimePrimers	5'-TTAAAAACCTGGATCGGAACCAA-3' 5'-GCATTAGCTTCAGATTTACGGGT-3'
β-Actin	RealTimePrimers	5'-CTCTTCCAGCCTTCCTTCCT-3' 5'-TGCTAGGGCTGTGATCTCCT-3'

### Statistical analysis

To test statistical significance, one-way ANOVA was used to compare multiple comparison groups followed by Tukey’s post-hoc test, and Student’s unpaired t-test was used to compare 2 independent groups. GraphPad Prism software, version 10.1 (GraphPad Software, USA) was used. *P* value ≤ 0.05 was considered statistically significant.

## Supplementary Information

Additional file 1: Figure S1. Principle Components Analysis of all lung fibrotic disease groups. PCA showing (A) COVID-ARDS group compared to IPF alone and IPF with COVID history groups combined (B) IPF with COVID history group compared to COVID-ARDS group (C) IPF with COVID history group compared to IPF alone group. N = 3 for each group (COVID-ARDS, IPF alone, and IPF with COVID history). Figure S2. Baseline mRNA expression levels in mock or empty virus treated MLE-12 cells expressing IGFBP2, IGF1 and IGF2. Data are representative of minimum of 3 independent experiments. Data are presented as mean ± SEM. *** P <0.001 Student Unpaired t-test.

Additional file 2: Table S1: Demographic Information. Baseline demographic and clinical characteristics of end-stage lung disease patients. Table S2: Differentially expressed genes in isolated AEC2 cells. List of all differentially expressed genes in AEC2 cells of patients with COVID-ARDS compared to rest of the lung disease groups combined (IPF alone and IPF with history of moderate COVID). *p < 0.05. Table S3: Differentially expressed genes in isolated AEC2 cells. List of all differentially expressed genes in AEC2 cells of patients with IPF with history of moderate COVID compared to COVID-ARDS group. *p < 0.05. Table S4: Differentially expressed genes in isolated AEC2 cells. List of all differentially expressed genes in AEC2 cells of patients with IPF with history of moderate COVID compared to IPF alone group. *p < 0.05. Table S5: Gene ontology (GO) term enrichment of the top 20 differentially expressed genes in AEC2 cells of patients with IPF along with history of moderate COVID and COVID-ARDS, IPF alone groups.

## Data Availability

Please contact corresponding author for data requests.
