# Supplementary material for: Alveolar epithelial type 2 cell specific loss of IGFBP2 activates inflammation in COVID-19
Source: Respir Res. 2025 Mar 22;26:111. doi: 10.1186/s12931-025-03187-9 (PMC11929192; doi:10.1186/s12931-025-03187-9)
Supplement: Supplementary file 1 — Additional file 1: Figure S1. Principle Components Analysis of all lung fibrotic disease groups. PCA showing (A) COVID-ARDS group compared to IPF alone and IPF with COVID history groups combined (B) IPF with COVID history group compared to COVID-ARDS group (C) IPF with COVID history group compared to IPF alone group. N = 3 for each group (COVID-ARDS, IPF alone, and IPF with COVID history). Figure S2. Baseline mRNA expression levels in mock or empty virus treated MLE-12 cells expressing IGFBP2, IGF1 and IGF2. Data are representative of minimum of 3 independent experiments. Data are presented as mean ± SEM. *** P <0.001 Student Unpaired t-test. [file 12931_2025_3187_MOESM1_ESM.docx]

Supplementary Information

**Alveolar Type 2 Epithelial Specific Loss of IGFBP2 Activates Inflammation in COVID-19**

Valentina Pujadas, Chiahsuan Chin, Narendra V. Sankpal, James Buhrmaster, Ashwini Arjuna, Rajat Walia, Michael A. Smith, Oliver Eickelberg, Ross M. Bremner, Thalachallour Mohanakumar^,^ and Angara Sureshbabu^*^

**Figure S1**


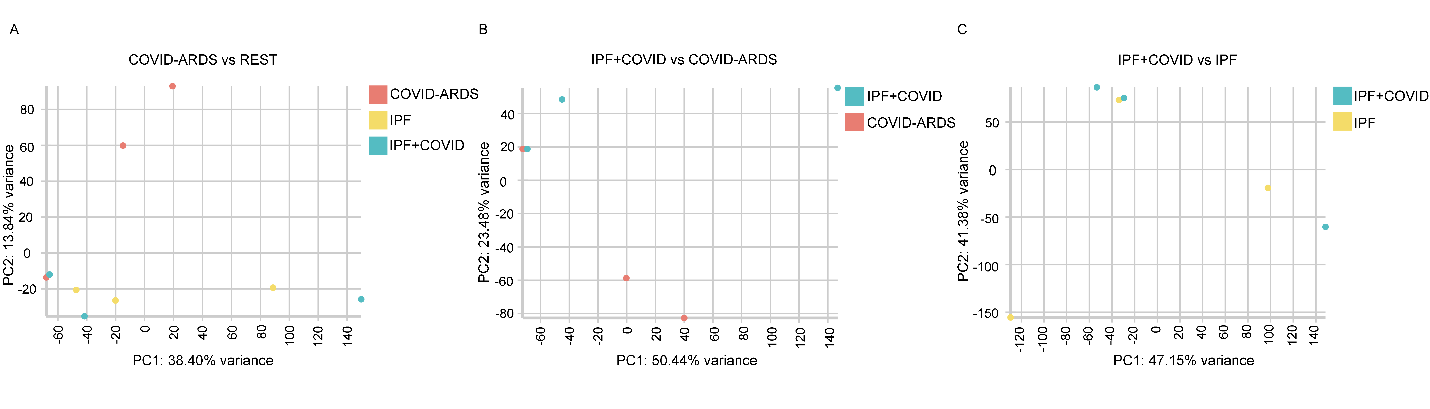


**Supplementary Figure Legends**

**Figure S1.** Principle Components Analysis of all lung fibrotic disease groups. PCA showing (A) COVID-ARDS group compared to IPF alone and IPF with COVID history groups combined (B) IPF with COVID history group compared to COVID-ARDS group (C) IPF with COVID history group compared to IPF alone group. N = 3 for each group (COVID-ARDS, IPF alone, and IPF with COVID history).

**Figure S2**


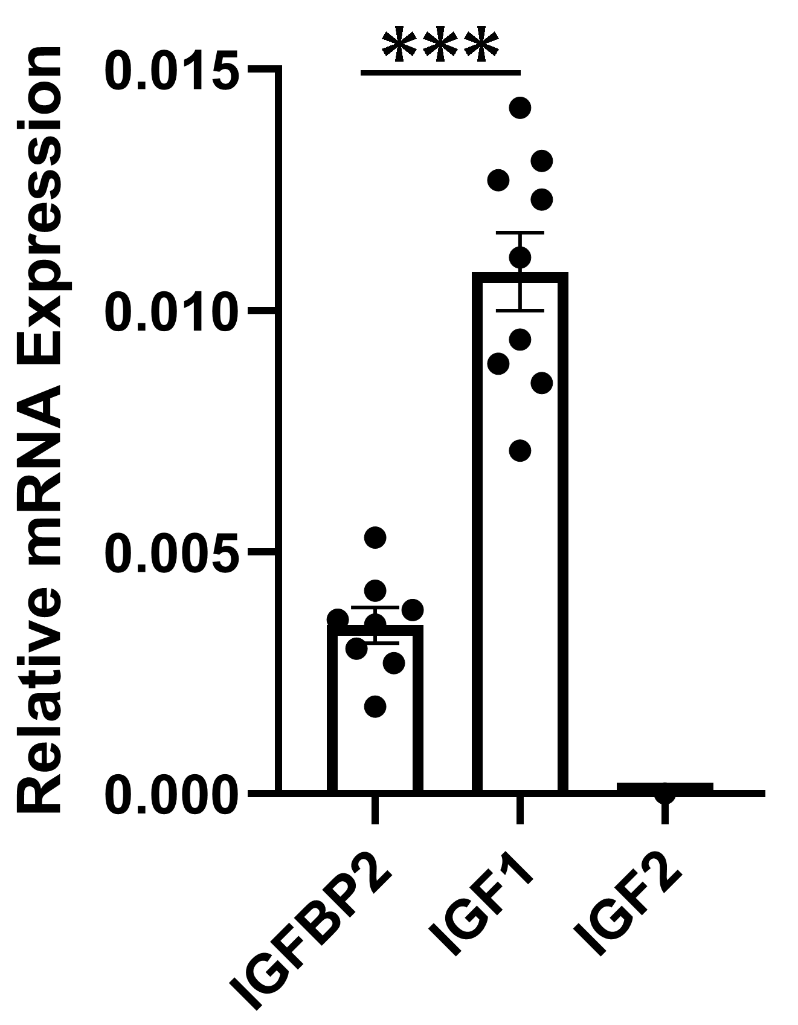


**Figure S2.** Baseline mRNA expression levels in mock or empty virus treated MLE-12 cells expressing IGFBP2, IGF1 and IGF2. Data are representative of minimum of 3 independent experiments. Data are presented as mean ± SEM. *** P <0.001 Student Unpaired t-test.
