# Supplementary material for: Alveolar epithelial type 2 cell specific loss of IGFBP2 activates inflammation in COVID-19
Source: Respir Res. 2025 Mar 22;26:111. doi: 10.1186/s12931-025-03187-9 (PMC11929192; doi:10.1186/s12931-025-03187-9)
Supplement: Supplementary file 2 — Additional file 2: Table S1: Demographic Information. Baseline demographic and clinical characteristics of end-stage lung disease patients. Table S2: Differentially expressed genes in isolated AEC2 cells. List of all differentially expressed genes in AEC2 cells of patients with COVID-ARDS compared to rest of the lung disease groups combined (IPF alone and IPF with history of moderate COVID). *p < 0.05. Table S3: Differentially expressed genes in isolated AEC2 cells. List of all differentially expressed genes in AEC2 cells of patients with IPF with history of moderate COVID compared to COVID-ARDS group. *p < 0.05. Table S4: Differentially expressed genes in isolated AEC2 cells. List of all differentially expressed genes in AEC2 cells of patients with IPF with history of moderate COVID compared to IPF alone group. *p < 0.05. Table S5: Gene ontology (GO) term enrichment of the top 20 differentially expressed genes in AEC2 cells of patients with IPF along with history of moderate COVID and COVID-ARDS, IPF alone groups. [file 12931_2025_3187_MOESM2_ESM.docx]

Table S1: Demographic Information. Baseline demographic and clinical characteristics of end-stage lung disease patients.

| Characteristics | Donor^a^  n=6 | COVID- ARDS  n=3 | IPF  n=5 | IPF-COVID^b^  n=5 |
| --- | --- | --- | --- | --- |
| Demographics | | | | |
| Age, median (IQR) | 66.5 (58.25-80.5) | 43  (36-71) | 72  (69-73) | 64  (60.50-72.50) |
| Sex (female/male) | 2/4 | 0/3 | 1/4 | 1/4 |
| Ethnicity (C/H/A) | 6/0/0 | 2/1/0 | 4/1/0 | 3/1/1 |
| BMI, median (IQR) | 20.47 (19.68-21.25) | 28.10  (28-32.81) | 28.9 (23.57-29.93) | 25.24 (20.50-27.78) |
| Chronic Comorbidities | | | | |
| Type 2 diabetes (Yes/No) | 2/4 | 0/3 | 1/4 | 0/5 |
| Smoking history (Yes/No) | 0/6 | 0/3 | 1/4 | 3/2 |

^a^1 Unknown donor. ^b^IPF with moderate COVID history. Abbreviations: ARDS, acute respiratory distress syndrome; A, Asian; BMI, body mass index; C, Caucasian; COVID, coronavirus disease; H, Hispanic; IPF, idiopathic pulmonary fibrosis;

Table S2: Differentially expressed genes in isolated AEC2 cells. List of all differentially expressed genes in AEC2 cells of patients with COVID-ARDS compared to rest of the lung disease groups combined (IPF alone and IPF with history of moderate COVID). *p < 0.05

| **Gene Symbol** | **log2FoldChange** | **p-value** |
| --- | --- | --- |
| SNORD89 | 3.714465938 | 1.13931E-06 |
| MIR627 | 3.713232227 | 0.013977646 |
| MIR320C1 | 3.448739859 | 0.007347159 |
| SNORD95 | 3.193954138 | 0.001299355 |
| SNORD1C | 3.172270318 | 0.002175227 |
| PTPRD-AS1 | 3.137110439 | 0.011361138 |
| SNORD41 | 3.045102212 | 2.65397E-06 |
| OR10H3 | 3.017517751 | 0.050693198 |
| MIR3173 | 3.000289664 | 0.048981336 |
| ACR | 2.863835133 | 0.012189046 |
| C8orf49 | 2.675705255 | 0.038006382 |
| SFTPD | 2.675586409 | 0.006646439 |
| SNORD33 | 2.620476257 | 0.000223702 |
| UBB | 2.512097337 | 0.00430011 |
| EMP3 | 2.455153451 | 0.00540372 |
| TCEAL2 | 2.431766262 | 0.014419754 |
| HSD17B6 | 2.385752129 | 0.026141276 |
| LSM4 | 2.369912842 | 0.000156233 |
| PALM3 | 2.349406515 | 0.003247372 |
| SSTR5-AS1 | 2.339926327 | 0.000292068 |
| C4BPA | 2.325519357 | 0.027411504 |
| STXBP5-AS1 | 2.272411973 | 0.009866081 |
| LOC101927587 | 2.269184348 | 0.04572977 |
| SFTA1P | 2.253494937 | 0.002666105 |
| CHAC2 | 2.244684063 | 0.035908557 |
| NHLH1 | 2.244666702 | 0.012226622 |
| MIR7107 | 2.23538737 | 0.050829438 |
| NAP1L5 | 2.234125848 | 0.002452176 |
| CGNL1 | 2.232811502 | 0.000965379 |
| JUND | 2.215206054 | 0.000172947 |
| SFRP5 | 2.167270517 | 0.021775444 |
| THEG5 | 2.130246851 | 0.006830038 |
| IL21-AS1 | 2.11754439 | 0.017560452 |
| SNORD57 | 2.103819117 | 0.022461387 |
| CCDC70 | 2.096449211 | 0.040612946 |
| CA11 | 2.06280278 | 0.006852832 |
| INHBA | 2.046715982 | 0.018727634 |
| NAT2 | 2.031673105 | 0.020274469 |
| BEX2 | 2.019226895 | 0.002695055 |
| NUPR1 | 1.987533547 | 0.013140024 |
| HDAC6 | 1.987323348 | 0.000445171 |
| NDUFA4 | 1.954956047 | 0.002106154 |
| SLC15A3 | 1.953786765 | 0.002964235 |
| USP30-AS1 | 1.94842056 | 0.02377273 |
| RNA5-8S5 | 1.925790616 | 0.010973691 |
| FAM159B | 1.925346617 | 0.032483111 |
| MARCO | 1.917052989 | 0.036471698 |
| COA6 | 1.912528682 | 0.008374147 |
| SZRD1 | 1.899353565 | 0.000183859 |
| RND1 | 1.889235147 | 0.019864453 |
| APTR | 1.857647017 | 0.000778365 |
| MEX3A | 1.834172461 | 0.013177673 |
| TMEM74B | 1.822425436 | 0.028210961 |
| ZNF628 | 1.81656749 | 0.034526001 |
| PCED1B-AS1 | 1.789554818 | 0.031444053 |
| ELOVL5 | 1.781256212 | 0.000535016 |
| GTF2H2C | 1.75641217 | 0.021823395 |
| KCNC3 | 1.746868168 | 0.008617987 |
| SFTA3 | 1.74411792 | 0.037882139 |
| LINC01354 | 1.742890619 | 0.0392537 |
| FBXL19 | 1.74222526 | 0.025399209 |
| GJA1 | 1.74137583 | 0.027440425 |
| GTF2H2C_2 | 1.729952046 | 0.024596484 |
| TRIM52-AS1 | 1.720743002 | 0.019095762 |
| TMEM238 | 1.718635666 | 0.030986945 |
| SAP30 | 1.713664151 | 0.020893434 |
| NDUFA12 | 1.685920982 | 0.023904089 |
| NRGN | 1.68387286 | 0.01757261 |
| C1QC | 1.682196772 | 0.035648674 |
| C19orf68 | 1.679996159 | 0.000269282 |
| KIAA0408 | 1.675296373 | 0.035170379 |
| PPP1R1B | 1.670616031 | 0.017704159 |
| LOC729867 | 1.668673926 | 0.003168016 |
| ERMN | 1.66391416 | 0.04551115 |
| NDUFA6 | 1.662101743 | 0.00678214 |
| GFER | 1.651377508 | 0.023652844 |
| BRIX1 | 1.632793952 | 0.020032996 |
| SUGCT | 1.618608922 | 0.024709637 |
| NR1H2 | 1.60912944 | 0.000310218 |
| DNAJB5 | 1.608858818 | 0.032681392 |
| FBXW4 | 1.608167914 | 0.00020207 |
| IL11RA | 1.608083193 | 0.012255218 |
| C6orf57 | 1.594405988 | 0.036024114 |
| B3GALT6 | 1.592343625 | 0.016136247 |
| C1QB | 1.586403699 | 0.038371239 |
| FHDC1 | 1.582291943 | 0.004722386 |
| LINC01011 | 1.578320814 | 0.049176528 |
| ATP13A4-AS1 | 1.572957505 | 0.027931404 |
| AEBP2 | 1.564515847 | 0.008072293 |
| NFATC4 | 1.562816539 | 0.006912697 |
| MRPS33 | 1.562694855 | 0.019494171 |
| NPNT | 1.549450718 | 0.014245809 |
| TAF15 | 1.54232298 | 0.050098206 |
| DLD | 1.541440142 | 0.013082785 |
| FRA10AC1 | 1.536151163 | 0.002664577 |
| NRAS | 1.535701761 | 0.042580412 |
| DOK2 | 1.530998497 | 0.013664367 |
| SCARNA21 | 1.520638594 | 0.039476095 |
| FBL | 1.520174798 | 0.005483496 |
| MIR3917 | 1.51848776 | 0.03651219 |
| SLC39A8 | 1.516517814 | 0.009330911 |
| FADS2 | 1.499409539 | 0.001231105 |
| SNHG22 | 1.499404972 | 0.009321801 |
| S100A14 | 1.495817886 | 0.023795331 |
| RAB5C | 1.491884886 | 0.010364073 |
| ZBTB47 | 1.491376309 | 0.010070498 |
| PARM1 | 1.490955014 | 0.002694676 |
| PTRF | 1.489711298 | 0.007495864 |
| LRRK2 | 1.483924919 | 0.014884895 |
| RSBN1L | 1.481928941 | 0.007246186 |
| PCED1B | 1.476311499 | 0.046209771 |
| LYAR | 1.471143731 | 0.017088278 |
| LYRM1 | 1.470869808 | 0.007119505 |
| ZNF787 | 1.469505722 | 0.038479873 |
| INSIG2 | 1.467881669 | 0.031551463 |
| CCM2 | 1.46686959 | 0.025270226 |
| SLC25A10 | 1.463837985 | 0.014315238 |
| CADM1 | 1.462386084 | 0.025039797 |
| SKIV2L2 | 1.454312811 | 0.00620155 |
| RNF121 | 1.439826179 | 0.021278519 |
| GMFB | 1.439503616 | 0.050942149 |
| RHOBTB2 | 1.418535892 | 0.010258951 |
| SASH1 | 1.413828207 | 0.003104327 |
| CEP70 | 1.411390179 | 0.012011051 |
| LAMTOR1 | 1.409687587 | 0.012385677 |
| PYCR2 | 1.401347109 | 0.018133998 |
| PGRMC2 | 1.40058505 | 0.007081829 |
| DEK | 1.393852433 | 0.050012648 |
| PXMP4 | 1.388728349 | 0.016976769 |
| HIRIP3 | 1.38723348 | 0.005632025 |
| NDUFAF2 | 1.37834142 | 0.032505119 |
| TRIM44 | 1.375974562 | 0.023986692 |
| ERP29 | 1.367751364 | 0.023719071 |
| PRRT2 | 1.366822249 | 0.006264213 |
| PGBD3 | 1.366271483 | 0.009625371 |
| GAR1 | 1.36589037 | 0.005246024 |
| TSPAN7 | 1.36549678 | 0.04813565 |
| PDCL3 | 1.364878436 | 0.030400914 |
| ARL5A | 1.361062507 | 0.032139115 |
| LPCAT1 | 1.359547248 | 0.036598473 |
| RAB21 | 1.355664245 | 0.019285726 |
| AK1 | 1.354995383 | 0.002812257 |
| VPS26A | 1.353462279 | 0.024327533 |
| MTA2 | 1.352956586 | 0.006305908 |
| LOC100652999 | 1.350515463 | 0.003507267 |
| TRIM41 | 1.349254316 | 0.009295389 |
| SMARCA5-AS1 | 1.345254512 | 0.02285396 |
| PDHA1 | 1.34431939 | 0.03360686 |
| SEC62 | 1.338305097 | 0.050183483 |
| FLJ23867 | 1.337702225 | 0.042896045 |
| ZNF503 | 1.328577552 | 0.046630542 |
| MVK | 1.321181344 | 0.026100261 |
| CPM | 1.319555478 | 0.048574475 |
| ZBTB7A | 1.309965169 | 0.019012692 |
| PBXIP1 | 1.308851515 | 0.004897692 |
| GLS | 1.308792139 | 0.000814841 |
| LENG1 | 1.308262623 | 0.027364063 |
| ENY2 | 1.305809193 | 0.033044353 |
| CCDC23 | 1.296925075 | 0.03990837 |
| NAB2 | 1.284816426 | 0.012439149 |
| JAZF1 | 1.273658966 | 0.041684046 |
| TAPT1 | 1.267596944 | 0.004432654 |
| SETD3 | 1.266952451 | 0.02833452 |
| CDC42EP1 | 1.254717757 | 0.047295647 |
| NDNF | 1.249218692 | 0.033633436 |
| RNA45S5 | 1.241392312 | 0.009595216 |
| ICAM1 | 1.240140777 | 0.033941981 |
| IRX3 | 1.234990722 | 0.028077093 |
| MAZ | 1.229933279 | 0.013969035 |
| ATP11A | 1.22818331 | 0.047997898 |
| HDDC2 | 1.211173239 | 0.017420791 |
| TEX261 | 1.207622257 | 0.013674487 |
| BCAM | 1.197849108 | 0.025627513 |
| TMA16 | 1.197181006 | 0.034710628 |
| FAM136A | 1.164798493 | 0.038966892 |
| DCTN3 | 1.162881925 | 0.044112642 |
| PPP2R2A | 1.159082565 | 0.031632921 |
| LARP4 | 1.157963533 | 0.034225438 |
| ELK1 | 1.157960673 | 0.042836759 |
| PDHB | 1.157738652 | 0.035086057 |
| DBN1 | 1.155137503 | 0.026837343 |
| NFIX | 1.15326622 | 0.009786241 |
| MAF | 1.148613706 | 0.046535867 |
| UBFD1 | 1.148083599 | 0.027749533 |
| CADM4 | 1.144715284 | 0.049338046 |
| ERF | 1.136481762 | 0.019104678 |
| AES | 1.129412048 | 0.034000838 |
| SLC31A1 | 1.129206801 | 0.04661744 |
| TMEM41B | 1.115976456 | 0.02560794 |
| GTF2F1 | 1.108253219 | 0.037892244 |
| MAP2K7 | 1.106475601 | 0.011298162 |
| PLD3 | 1.106350777 | 0.028942767 |
| PSMC3 | 1.105125531 | 0.042759933 |
| ACADSB | 1.103745253 | 0.027385168 |
| UXS1 | 1.09897733 | 0.047093098 |
| CITED2 | 1.092967027 | 0.044590929 |
| RAD23A | 1.084003265 | 0.045993634 |
| MMP24-AS1 | 1.080018384 | 0.028103831 |
| SLC39A1 | 1.078472283 | 0.012336411 |
| LIN7C | 1.076716891 | 0.037924319 |
| CENPB | 1.073879811 | 0.015506712 |
| MAX | 1.071408939 | 0.045131933 |
| ZNF444 | 1.065186414 | 0.025066115 |
| ZBTB4 | 1.063057291 | 0.008852035 |
| ECHDC1 | 1.056456919 | 0.049648572 |
| SLC41A1 | 1.054143756 | 0.03788419 |
| NFKBIB | 1.047890899 | 0.032574952 |
| EIF4E2 | 1.044800154 | 0.019435686 |
| CDV3 | 1.044465457 | 0.04849425 |
| IMPA2 | 1.035944345 | 0.030724754 |
| PLCB2 | 1.033066662 | 0.048239984 |
| FAM71E1 | 1.030523574 | 0.029125264 |
| PPP1R7 | 1.011513826 | 0.049995756 |
| EFNB2 | 1.009349147 | 0.042615933 |
| TNS1 | 0.978217512 | 0.036861983 |
| DEDD | 0.959438777 | 0.04524808 |
| MGLL | 0.958607825 | 0.022735862 |
| LPCAT3 | 0.954454142 | 0.042183335 |
| PBX1 | 0.952403241 | 0.010350617 |
| PDCD7 | 0.948318115 | 0.044278848 |
| LDB1 | 0.947603856 | 0.016210676 |
| ZC3H4 | 0.921465054 | 0.023071758 |
| ZNF316 | 0.919668362 | 0.010514962 |
| BAIAP2-AS1 | 0.91188797 | 0.015554309 |
| WIPF2 | 0.909769419 | 0.023282562 |
| PHF23 | 0.891838842 | 0.04778651 |
| NACC1 | 0.88716554 | 0.02022781 |
| ATF7 | 0.886150573 | 0.017973631 |
| KDELR1 | 0.882593615 | 0.042531373 |
| CBX4 | 0.8814828 | 0.029171742 |
| ATN1 | 0.877412749 | 0.031849737 |
| PRR12 | 0.869455489 | 0.047483448 |
| RAB1B | 0.859205234 | 0.050463944 |
| VAMP2 | 0.853469095 | 0.050380343 |
| KAT6B | 0.812267475 | 0.050031643 |
| NFIC | 0.793178206 | 0.019414303 |
| PPP1R26 | 0.791282166 | 0.029119575 |
| BCL9L | 0.765062782 | 0.028073474 |
| VPS53 | 0.745491713 | 0.039294317 |
| KMT2D | 0.67576255 | 0.024391898 |
| FMN1 | -0.807236771 | 0.047696746 |
| CHFR | -0.823028154 | 0.05026711 |
| MAN2A2 | -0.881883992 | 0.047482338 |
| PLXNA1 | -0.933787509 | 0.032195986 |
| VAV1 | -0.978842541 | 0.043615796 |
| UTP20 | -0.982501888 | 0.043111112 |
| SIPA1L2 | -1.004466648 | 0.04381615 |
| ATP12A | -1.004678589 | 0.033358826 |
| DNAJC16 | -1.005553758 | 0.023527569 |
| NADSYN1 | -1.039505909 | 0.049762685 |
| ITGB4 | -1.055757493 | 0.03558763 |
| UNC93B1 | -1.0942304 | 0.035778941 |
| BRCA1 | -1.107665877 | 0.046448862 |
| AMPD3 | -1.125292212 | 0.033102406 |
| ATP2A3 | -1.148810379 | 0.039317982 |
| USP37 | -1.155117238 | 0.037345708 |
| PITPNC1 | -1.162619136 | 0.049970865 |
| PKP2 | -1.177866567 | 0.047832461 |
| SYNJ2 | -1.187693466 | 0.035042172 |
| SRGAP1 | -1.203005418 | 0.034576986 |
| CHST15 | -1.206655002 | 0.035116177 |
| TTL | -1.218222863 | 0.044404506 |
| DGKH | -1.239314036 | 0.03607663 |
| SRGAP3 | -1.249277186 | 0.012792333 |
| PIK3CG | -1.252436843 | 0.039039065 |
| LGR4 | -1.254565427 | 0.047177343 |
| PREX1 | -1.25771959 | 0.044846641 |
| IPO4 | -1.259226013 | 0.033693068 |
| MUC16 | -1.263866603 | 0.022367952 |
| OAT | -1.26487489 | 0.030230472 |
| SETBP1 | -1.267602211 | 0.034346852 |
| ENTPD1 | -1.271368398 | 0.013135864 |
| PHC1 | -1.275139576 | 0.043629719 |
| RHBDF2 | -1.286638607 | 0.04447067 |
| TRIM29 | -1.3077556 | 0.02325963 |
| IL16 | -1.310481968 | 0.017302736 |
| FANCI | -1.313282591 | 0.035407051 |
| PSTPIP1 | -1.315729185 | 0.015967248 |
| INPP4B | -1.326506408 | 0.044496177 |
| PPP1R16B | -1.329247477 | 0.048495892 |
| PKD1P6 | -1.331605002 | 0.038958456 |
| AOAH | -1.333173759 | 0.04061929 |
| GMDS | -1.358914441 | 0.046234489 |
| IRF1 | -1.361696808 | 0.047800324 |
| FMNL1 | -1.376744184 | 0.007585194 |
| LOC399491 | -1.377187304 | 0.020042894 |
| NWD1 | -1.387706945 | 0.050429065 |
| DCLRE1C | -1.387867976 | 0.047862357 |
| ACAP1 | -1.393833189 | 0.033021043 |
| KIAA0825 | -1.397077345 | 0.040741442 |
| SIGLEC9 | -1.401388842 | 0.029697446 |
| RIPK4 | -1.402695266 | 0.045584524 |
| C20orf96 | -1.403468699 | 0.042770274 |
| SLC37A2 | -1.409094627 | 0.01648752 |
| GEN1 | -1.415521419 | 0.04450172 |
| C21orf58 | -1.424279015 | 0.029448061 |
| KLHL21 | -1.433110067 | 0.01491877 |
| LILRB2 | -1.433122958 | 0.011908612 |
| BCKDK | -1.4356635 | 0.047826101 |
| RB1 | -1.437912614 | 0.025306551 |
| EXOC6 | -1.446814404 | 0.034307546 |
| SH3TC1 | -1.451338331 | 0.038882661 |
| LRRC8B | -1.451482941 | 0.026051147 |
| TPCN2 | -1.452281971 | 0.037646839 |
| NFKBIE | -1.457527231 | 0.026180516 |
| EPHB2 | -1.458986797 | 0.050653959 |
| GCLC | -1.462257545 | 0.043033013 |
| ATP13A1 | -1.465751088 | 0.019809802 |
| ADAM8 | -1.468467123 | 0.029927121 |
| PSMB10 | -1.474837999 | 0.050241926 |
| ATP13A2 | -1.488103566 | 0.02864357 |
| WDR54 | -1.490903348 | 0.045526164 |
| AKAP6 | -1.494176811 | 0.03336527 |
| KIAA1551 | -1.494192549 | 0.043400721 |
| SLFN12 | -1.501717874 | 0.043484723 |
| USP31 | -1.505678442 | 0.017814062 |
| WDR35 | -1.505970637 | 0.037714421 |
| ITGB8 | -1.508738811 | 0.011999661 |
| BCL2 | -1.513172661 | 0.039641057 |
| FAM45B | -1.513802748 | 0.040429485 |
| LILRB4 | -1.515123184 | 0.029887441 |
| NARFL | -1.527190747 | 0.031161019 |
| CFAP61 | -1.529265678 | 0.02732716 |
| NPAS2 | -1.531173333 | 0.044753438 |
| STAG3L2 | -1.542485735 | 0.049658081 |
| RHPN2 | -1.554708035 | 0.024459366 |
| TPST2 | -1.557147663 | 0.017587476 |
| FAM86A | -1.562161071 | 0.038492354 |
| CDH26 | -1.566809691 | 0.050889868 |
| WDR90 | -1.567659507 | 0.046172072 |
| FAIM3 | -1.571503907 | 0.037555665 |
| FAM65B | -1.575108108 | 0.048912799 |
| SLC25A12 | -1.581525304 | 0.027609197 |
| DDX56 | -1.583601162 | 0.043258132 |
| ARHGEF37 | -1.586680934 | 0.011623059 |
| GBP6 | -1.590139747 | 0.035983683 |
| ARNTL2 | -1.590292133 | 0.01804071 |
| VOPP1 | -1.592702025 | 0.029375736 |
| SERAC1 | -1.59313609 | 0.049115585 |
| LOC440434 | -1.609849724 | 0.031132798 |
| SIGLEC7 | -1.611737637 | 0.044090097 |
| GPR110 | -1.612372976 | 0.019671686 |
| LMLN | -1.615313097 | 0.026039753 |
| NAAA | -1.624934835 | 0.043781346 |
| PXK | -1.624937082 | 0.007189495 |
| PLEKHH1 | -1.629551468 | 0.037566992 |
| MTMR2 | -1.637539055 | 0.03402421 |
| IL5RA | -1.638717666 | 0.018851908 |
| TRAF3IP3 | -1.638953836 | 0.013842755 |
| KIAA1456 | -1.644213538 | 0.032460281 |
| KCNN4 | -1.644318034 | 0.049095567 |
| LOC200772 | -1.648950624 | 0.032348132 |
| TCTN1 | -1.658929545 | 0.021363026 |
| GUSBP1 | -1.661245987 | 0.050995735 |
| LOXL4 | -1.68429776 | 0.011381731 |
| EEPD1 | -1.692766644 | 0.018222289 |
| TMEM231 | -1.696203161 | 0.044983886 |
| LTB4R2 | -1.701109626 | 0.042812934 |
| TNNI2 | -1.701728582 | 0.010221665 |
| CARF | -1.712395777 | 0.034124509 |
| CDYL2 | -1.716235022 | 0.046745402 |
| ICA1L | -1.720538145 | 0.030431238 |
| SCAI | -1.721928016 | 0.041533069 |
| LRMP | -1.724964467 | 0.010681287 |
| RAB36 | -1.734890021 | 0.050607257 |
| CDS1 | -1.744494003 | 0.012923923 |
| PRKX | -1.761882718 | 0.004564419 |
| KNTC1 | -1.766066634 | 0.018263485 |
| SLC22A4 | -1.769231492 | 0.037654923 |
| PRKCB | -1.778147138 | 0.001258235 |
| ATRIP | -1.779806131 | 0.034097056 |
| TSPAN8 | -1.807806797 | 0.050898494 |
| NSMF | -1.812898265 | 0.038331781 |
| FAM198B | -1.81810607 | 0.003688352 |
| HDAC9 | -1.826601591 | 0.005347336 |
| MXD1 | -1.833921444 | 0.0377429 |
| GNAL | -1.834514261 | 0.041369037 |
| FRMD3 | -1.836246825 | 0.050822372 |
| TUBA4A | -1.83841445 | 0.026064069 |
| IPPK | -1.838541577 | 0.041764327 |
| AK8 | -1.838725629 | 0.035752348 |
| ARHGAP42 | -1.839756246 | 0.018093506 |
| CSMD1 | -1.849531521 | 0.044127476 |
| TGFA | -1.859264178 | 0.03271034 |
| KMO | -1.860025646 | 0.007805734 |
| HPSE | -1.860779476 | 0.028092818 |
| RAD52 | -1.862091349 | 0.048780942 |
| BRCA2 | -1.863382169 | 0.032618024 |
| CDKN2A | -1.865705062 | 0.04370519 |
| EML6 | -1.870061177 | 0.01881969 |
| FAT2 | -1.889227869 | 0.00430439 |
| LRRN1 | -1.893883602 | 0.037337353 |
| GRIN3B | -1.894709074 | 0.028632714 |
| CR1 | -1.904089569 | 0.005145289 |
| CD247 | -1.915102346 | 0.047442296 |
| ADAM19 | -1.915317461 | 0.003001495 |
| RSPH9 | -1.926078392 | 0.032975655 |
| CYTIP | -1.934961775 | 0.02167159 |
| SEMA3A | -1.938536623 | 0.042363949 |
| PDE1B | -1.944458163 | 0.034619094 |
| LTBP2 | -1.945939316 | 0.027288155 |
| FRMPD2 | -1.95243038 | 0.03102751 |
| PSMD5-AS1 | -1.954441532 | 0.04101893 |
| DCLK1 | -1.955934726 | 0.012995012 |
| ANXA8L1 | -1.956998956 | 0.043735408 |
| POLE4 | -1.965328956 | 0.037146053 |
| RRN3P2 | -1.966100242 | 0.032178113 |
| LCK | -1.968972557 | 0.0391563 |
| EMR2 | -1.969318021 | 0.002091763 |
| TEKT4 | -1.973518761 | 0.015977883 |
| SYNE3 | -1.975889869 | 0.008533986 |
| KIAA0319 | -1.977388893 | 0.009318891 |
| FAM118A | -1.977840481 | 0.009716135 |
| ENPP2 | -1.98003328 | 0.025197129 |
| TMEM180 | -1.982033898 | 0.049908501 |
| SLC9A3 | -1.985405359 | 0.050684483 |
| NLRC3 | -1.987398973 | 0.017471377 |
| BCO2 | -1.989648735 | 0.022989974 |
| GABRP | -1.990815818 | 0.044104253 |
| DCDC5 | -1.992923706 | 0.024809376 |
| CNTNAP3 | -2.00270436 | 0.037763819 |
| CXCR4 | -2.004016561 | 0.03754845 |
| OPN3 | -2.00631878 | 0.01739889 |
| LEKR1 | -2.017967327 | 0.030984557 |
| WDR25 | -2.021522854 | 0.043517443 |
| PIGV | -2.021560618 | 0.050885293 |
| IL1B | -2.024294226 | 0.030689553 |
| BNC2 | -2.026165264 | 0.047372271 |
| CHIT1 | -2.029626157 | 0.00842187 |
| STK32C | -2.032957357 | 0.044534516 |
| GNG10 | -2.033053363 | 0.033112602 |
| CLEC19A | -2.039473747 | 0.050422964 |
| CYP1B1 | -2.039741209 | 0.011044649 |
| S100A8 | -2.041133919 | 0.049226301 |
| MKNK1-AS1 | -2.054387093 | 0.016835202 |
| CCZ1 | -2.059816982 | 0.049799943 |
| PARD6G | -2.067678068 | 0.035636291 |
| ITGA4 | -2.072918464 | 0.006057018 |
| CTSK | -2.076917078 | 0.038287007 |
| F5 | -2.087854801 | 0.015198756 |
| ACBD7 | -2.091219643 | 0.032990388 |
| MORN5 | -2.098966729 | 0.033768803 |
| CAPSL | -2.106879612 | 0.046219142 |
| TMTC1 | -2.107393848 | 0.046931485 |
| AKAP5 | -2.111730297 | 0.044417868 |
| BEST1 | -2.114501456 | 0.001522895 |
| MROH9 | -2.121247069 | 0.023688954 |
| LMNTD1 | -2.121436354 | 0.043591241 |
| STRADB | -2.121880195 | 0.034601818 |
| ZKSCAN7 | -2.134810893 | 0.04015423 |
| UPK1B | -2.136257498 | 0.049970675 |
| FGF14 | -2.137841273 | 0.027811789 |
| SNCA | -2.141235356 | 0.035962815 |
| C1orf110 | -2.156686286 | 0.041233523 |
| CD22 | -2.163683379 | 0.010761196 |
| LOC399715 | -2.171550386 | 0.043088584 |
| RAPGEFL1 | -2.173722014 | 0.007433564 |
| PAOX | -2.182647511 | 0.047871025 |
| FBXL2 | -2.182719119 | 0.045958067 |
| FXYD6-FXYD2 | -2.193742814 | 0.040350057 |
| LOC101926935 | -2.20467474 | 0.013316957 |
| PTPRN2 | -2.205685996 | 0.009649453 |
| GJB3 | -2.215047366 | 0.029354908 |
| SNHG17 | -2.215604746 | 0.050697133 |
| TRPM2 | -2.222250402 | 0.011987985 |
| COL3A1 | -2.22286728 | 0.026830949 |
| SPHK1 | -2.235418995 | 0.030396952 |
| TRPC6 | -2.240490098 | 0.01124934 |
| DUSP2 | -2.241287548 | 0.025573688 |
| ALDH3A1 | -2.247280111 | 0.006100682 |
| CYP24A1 | -2.261300864 | 0.020388907 |
| FRMD6 | -2.273875262 | 0.050164316 |
| ZNF365 | -2.275099092 | 0.042821746 |
| KIAA1549L | -2.277504917 | 0.044963335 |
| TGFBR3 | -2.288020819 | 0.022798899 |
| DUS2 | -2.289827799 | 0.026218747 |
| PDZD4 | -2.298779017 | 0.010008529 |
| TMEM246 | -2.305246292 | 0.015238163 |
| THEMIS | -2.307978143 | 0.034465303 |
| KRT14 | -2.314428285 | 0.0358996 |
| MAT1A | -2.316641843 | 0.012970511 |
| B4GALT1-AS1 | -2.324702362 | 0.047022521 |
| MIR4435-1HG | -2.333228961 | 0.04458738 |
| ADAMTS14 | -2.341391584 | 0.019082295 |
| SERPINB3 | -2.346088173 | 0.043104792 |
| MAP1LC3B2 | -2.346602923 | 0.049863872 |
| CWH43 | -2.354693507 | 0.030701705 |
| PMEL | -2.362076267 | 0.05053588 |
| ALDH1L2 | -2.364244402 | 0.046241984 |
| CNTN5 | -2.375385868 | 0.04086739 |
| HPGD | -2.381257178 | 0.046666896 |
| ACTR5 | -2.389582255 | 0.038022142 |
| FAM86JP | -2.395296599 | 0.042796382 |
| CD96 | -2.407994387 | 0.047219343 |
| S100A2 | -2.411252871 | 0.012767816 |
| TPTEP1 | -2.415099062 | 0.050043992 |
| SLC2A6 | -2.419153688 | 0.045821828 |
| FBXW10 | -2.419759807 | 0.036762207 |
| MYOM2 | -2.430547426 | 0.036428113 |
| FCGR2C | -2.43102869 | 0.040269348 |
| CD1C | -2.431156681 | 0.048448633 |
| NTHL1 | -2.433187661 | 0.050978548 |
| HEY1 | -2.434457485 | 0.016400808 |
| LOC101927817 | -2.437835262 | 0.037268057 |
| AKR1C2 | -2.438704127 | 0.008033185 |
| FCGR1A | -2.440322546 | 0.013611622 |
| SLC16A9 | -2.440567775 | 0.042198768 |
| MEI1 | -2.441855556 | 0.042740826 |
| ADH7 | -2.446527291 | 0.027466306 |
| DENND2C | -2.45269604 | 0.031567596 |
| CAND2 | -2.45924668 | 0.026856359 |
| HAPLN3 | -2.462907089 | 0.029987979 |
| CD207 | -2.464169691 | 0.036984935 |
| FUT6 | -2.468741334 | 0.047593314 |
| ZNF19 | -2.472243921 | 0.018494811 |
| TGM1 | -2.477973807 | 0.030599114 |
| ARL6 | -2.487604247 | 0.022444247 |
| CSGALNACT1 | -2.495683966 | 0.016053348 |
| LPAR6 | -2.49697534 | 0.004997579 |
| ATP8B4 | -2.5006854 | 0.001758575 |
| ADCY2 | -2.502181406 | 0.042750989 |
| SCARF2 | -2.503253192 | 0.020884737 |
| ZAP70 | -2.515974482 | 0.017073134 |
| MKX | -2.522798924 | 0.021064788 |
| GCNT7 | -2.534333776 | 0.037680823 |
| SERPINB2 | -2.535520972 | 0.041994461 |
| ZNF474 | -2.53561359 | 0.0407968 |
| KRT16 | -2.540897039 | 0.027714635 |
| CCNI2 | -2.541162625 | 0.042181842 |
| M1AP | -2.546326767 | 0.043902237 |
| FDPSP2 | -2.54837119 | 0.00366654 |
| ZNF423 | -2.552378487 | 0.045188167 |
| LOC100505812 | -2.55813171 | 0.045204774 |
| TMPRSS4 | -2.5600427 | 0.000378705 |
| ADAMTS15 | -2.573550369 | 0.014434188 |
| ITGB3BP | -2.58404685 | 0.024507999 |
| STAMBPL1 | -2.586103409 | 0.046156403 |
| RIMBP2 | -2.586906767 | 0.043657973 |
| LOC101929653 | -2.587330561 | 0.031332584 |
| SGSM1 | -2.591610613 | 0.011085171 |
| APOBEC3A | -2.60723466 | 0.041315916 |
| FBLN2 | -2.619699516 | 0.025083171 |
| S100A9 | -2.619887644 | 0.017924859 |
| ADORA2A | -2.619974613 | 0.010644335 |
| TMEM133 | -2.620547738 | 0.048477006 |
| RAB42 | -2.621418392 | 0.03723147 |
| CCDC74B | -2.625684878 | 0.029990346 |
| PIF1 | -2.627576256 | 0.029749103 |
| CCNE1 | -2.632554121 | 0.047915949 |
| LINC00202-1 | -2.635722374 | 0.019484806 |
| LOC100129316 | -2.650880844 | 0.047319143 |
| NRON | -2.65242824 | 0.030169698 |
| ABCA6 | -2.652584872 | 0.034367876 |
| ABCD2 | -2.669855073 | 0.046280461 |
| GUCA1B | -2.671855449 | 0.028746739 |
| SV2A | -2.674876107 | 0.035965011 |
| CNTNAP3P2 | -2.675166409 | 0.040656867 |
| SELL | -2.68293136 | 0.010269796 |
| CNGA3 | -2.687073089 | 0.014523635 |
| CLDN16 | -2.687212041 | 0.031907302 |
| GCAT | -2.694629581 | 0.038425676 |
| CNTNAP3B | -2.699938533 | 0.03886599 |
| CCDC13-AS1 | -2.705891159 | 0.030528161 |
| LOC101927257 | -2.706353345 | 0.017370602 |
| BEST4 | -2.708476591 | 0.030335578 |
| CATSPERB | -2.71301557 | 0.022466074 |
| NCF1 | -2.732278748 | 0.008987407 |
| SYT8 | -2.737617484 | 0.000523377 |
| NLRP3 | -2.738305013 | 0.000357847 |
| TSPAN2 | -2.738750648 | 0.032004888 |
| RNF175 | -2.743124308 | 0.041879748 |
| ALG1L | -2.743966728 | 0.042767989 |
| DCLK2 | -2.748179709 | 0.027015367 |
| ADAMTS3 | -2.759635506 | 0.037107712 |
| ZBTB26 | -2.760689132 | 0.041696851 |
| PCDHB3 | -2.767985749 | 0.046869491 |
| SYCE2 | -2.777042713 | 0.040030076 |
| TRIP13 | -2.777471388 | 0.015381926 |
| TMEM200C | -2.781975159 | 0.048987975 |
| HNF4G | -2.785517732 | 0.009036707 |
| ACTA2 | -2.793638572 | 0.03345765 |
| KCNA6 | -2.795998764 | 0.035736728 |
| EGF | -2.797576672 | 0.033690472 |
| RAET1E | -2.799706526 | 0.032633168 |
| TUBA8 | -2.803687828 | 0.03706892 |
| ZIK1 | -2.803943026 | 0.035397967 |
| APOBEC3G | -2.807056719 | 0.018768847 |
| FAM90A1 | -2.81116463 | 0.040946264 |
| MROH2B | -2.814554345 | 0.045472339 |
| JAKMIP1 | -2.816438172 | 0.024426745 |
| GSG2 | -2.817285257 | 0.049072731 |
| TGM5 | -2.818167972 | 0.041242957 |
| BAALC | -2.822184256 | 0.032187862 |
| C14orf37 | -2.823610469 | 0.044072181 |
| SLC12A3 | -2.831897979 | 0.036138049 |
| MAPK11 | -2.838641134 | 0.023085683 |
| PTGIS | -2.838984892 | 0.026784661 |
| TMEM17 | -2.840907226 | 0.034517573 |
| ZNF569 | -2.847725799 | 0.018301322 |
| ILDR2 | -2.848978849 | 0.036413313 |
| ALG14 | -2.849965724 | 0.018595209 |
| EPS8L3 | -2.853291832 | 0.02434194 |
| LOC101928517 | -2.857049435 | 0.02318672 |
| CHADL | -2.858367302 | 0.007719992 |
| MKLN1-AS | -2.861825491 | 0.0458865 |
| PKD1L1 | -2.877330185 | 0.020266962 |
| TP53AIP1 | -2.881543866 | 0.024054537 |
| WDYHV1 | -2.884003921 | 0.044432646 |
| FSIP2 | -2.884635738 | 0.003093294 |
| DHRS4L1 | -2.88486235 | 0.02425191 |
| PAK3 | -2.887936212 | 0.040335941 |
| MB21D2 | -2.8889797 | 0.025283697 |
| NRCAM | -2.889220267 | 0.014439581 |
| SLC51A | -2.893376731 | 0.032091045 |
| XIRP1 | -2.895957088 | 0.038859182 |
| FLJ45079 | -2.899624921 | 0.035052049 |
| MMP11 | -2.904356961 | 0.04132935 |
| DNAJB7 | -2.914084712 | 0.045644439 |
| KCNS1 | -2.916080352 | 0.036179373 |
| CD48 | -2.920912736 | 0.01131815 |
| GNN | -2.922547946 | 0.016317276 |
| ZNF233 | -2.926486591 | 0.036902155 |
| LOC101927950 | -2.92815977 | 0.039557999 |
| LOC100147773 | -2.931686601 | 0.038618759 |
| GPBAR1 | -2.932532808 | 0.033704738 |
| PIWIL4 | -2.933346444 | 0.035170701 |
| TGFBR3L | -2.934090027 | 0.036442877 |
| COMMD8 | -2.936990958 | 0.039291074 |
| GJB2 | -2.940003082 | 0.033799392 |
| KLHL38 | -2.940072006 | 0.045020751 |
| MMP1 | -2.951112683 | 0.005356526 |
| DRICH1 | -2.95505715 | 0.028781042 |
| ENPP3 | -2.970322862 | 0.02977457 |
| TCN1 | -2.974890596 | 0.030770549 |
| ANKS4B | -2.975564453 | 0.048197084 |
| TFPI2 | -2.979533676 | 0.034699536 |
| TMC3 | -2.982203955 | 0.033586442 |
| CNIH2 | -2.983345729 | 0.025201118 |
| MCAM | -2.986321884 | 0.009351631 |
| IGSF22 | -2.988237326 | 0.009128639 |
| DIRC3 | -2.98937777 | 0.032535722 |
| ELOVL7 | -2.990446506 | 0.035259108 |
| GLT8D2 | -2.991612471 | 0.036963362 |
| TMEM47 | -2.992365349 | 0.031241744 |
| LINC00937 | -2.992783387 | 0.050114437 |
| GOLGA8H | -2.993777248 | 0.008403886 |
| LINC01301 | -2.993975446 | 0.043854219 |
| LIPG | -3.002996106 | 0.039908344 |
| RHBDD3 | -3.006614842 | 0.020048165 |
| CCDC150 | -3.009758562 | 0.044651601 |
| LOC101927446 | -3.011424728 | 0.043124603 |
| ADAMDEC1 | -3.022222526 | 0.016677522 |
| P2RX3 | -3.023554865 | 0.050508968 |
| SLC44A5 | -3.029699274 | 0.010775976 |
| GSTT1 | -3.029962813 | 0.030641331 |
| GPR45 | -3.030433789 | 0.042039363 |
| ATP6V1E2 | -3.033075554 | 0.039915087 |
| LOC102724297 | -3.033116159 | 0.026461935 |
| CSNK1G2-AS1 | -3.034900339 | 0.026751906 |
| GPR115 | -3.035896743 | 0.044630529 |
| GPR123 | -3.036678394 | 0.02920026 |
| RHOH | -3.043400463 | 0.004435167 |
| LAMA2 | -3.043415058 | 0.002627467 |
| LRRC63 | -3.048114697 | 0.032301845 |
| FLT3 | -3.050418506 | 0.017384147 |
| TMCC1-AS1 | -3.051277469 | 0.03145838 |
| FAM198A | -3.058314384 | 0.039806071 |
| KLHL30 | -3.058501993 | 0.037711472 |
| TRPC5 | -3.064526797 | 0.036908814 |
| SPAG4 | -3.066284222 | 0.013676683 |
| AFF2 | -3.067782613 | 0.017517951 |
| PDCD4-AS1 | -3.068240723 | 0.035154443 |
| SLC45A2 | -3.07426725 | 0.040582038 |
| GLUD2 | -3.078782028 | 0.039543363 |
| S100A12 | -3.080258373 | 0.038537619 |
| TMEM255B | -3.080520613 | 0.046432793 |
| FCGR2B | -3.086902258 | 0.011618141 |
| C1QTNF2 | -3.08775755 | 0.044918 |
| AOX1 | -3.089071728 | 0.03724827 |
| MEPE | -3.089738257 | 0.031549839 |
| EIF4E1B | -3.091548219 | 0.04477254 |
| TPSAB1 | -3.092100968 | 0.040086646 |
| ABCC2 | -3.096067909 | 0.040071799 |
| TXNDC8 | -3.098553495 | 0.013829418 |
| LOC101927865 | -3.099368396 | 0.048809514 |
| LOC102723344 | -3.109986685 | 0.04419511 |
| NR1I3 | -3.110136247 | 0.020711634 |
| C5orf60 | -3.110406469 | 0.046534444 |
| ACCSL | -3.11977999 | 0.044205259 |
| STAT4 | -3.122057056 | 0.011286649 |
| ANKRD33 | -3.126848483 | 0.040199321 |
| CCDC73 | -3.127938799 | 0.018678734 |
| CAMKV | -3.128018001 | 0.031743743 |
| ANTXRL | -3.134387482 | 0.044972371 |
| IPCEF1 | -3.134985317 | 0.000736136 |
| F2RL3 | -3.135396677 | 0.036742943 |
| C19orf83 | -3.139170394 | 0.034231551 |
| TMEM213 | -3.139386056 | 0.038404825 |
| SYT12 | -3.140620237 | 0.010678737 |
| SLC22A16 | -3.141064044 | 0.033658091 |
| CD226 | -3.154420276 | 0.011410149 |
| FAM24B-CUZD1 | -3.166769974 | 0.033701233 |
| WDR11-AS1 | -3.167016572 | 0.049336472 |
| PGF | -3.170999057 | 0.033799517 |
| CDKL4 | -3.17911301 | 0.043692552 |
| CALCRL | -3.180272007 | 0.030727794 |
| APOB | -3.180549019 | 0.025161271 |
| GPR83 | -3.180928091 | 0.039980684 |
| MGAT4EP | -3.182680468 | 0.047488483 |
| C17orf53 | -3.183834296 | 0.030902614 |
| GALNT13 | -3.184555563 | 0.045283646 |
| ABCB1 | -3.186943168 | 0.021472601 |
| CD244 | -3.189064198 | 0.017383704 |
| MMP16 | -3.190639162 | 0.044072871 |
| KRT6A | -3.190662626 | 0.005411556 |
| NOS2 | -3.194484733 | 0.04143536 |
| RAPGEF4 | -3.198458056 | 0.044211796 |
| NEURL3 | -3.198937124 | 0.044499108 |
| NYX | -3.204032824 | 0.043428017 |
| C19orf57 | -3.206855582 | 0.026344461 |
| LOC101929698 | -3.207481073 | 0.035795638 |
| SLC6A13 | -3.209851745 | 0.033427116 |
| SLC24A5 | -3.214706073 | 0.025881696 |
| ADAM7 | -3.2153708 | 0.042196622 |
| LOC101928861 | -3.217969545 | 0.037547059 |
| ISX | -3.223576514 | 0.032350004 |
| SHBG | -3.223591089 | 0.04164277 |
| SNAP25 | -3.224664669 | 0.049891178 |
| FAM21EP | -3.224835892 | 0.032291798 |
| POM121L4P | -3.225983915 | 0.036687208 |
| LIPN | -3.228051469 | 0.02907847 |
| LINC01506 | -3.228869691 | 0.026946997 |
| HOXB1 | -3.230040698 | 0.048412169 |
| PTX3 | -3.235297687 | 0.032440715 |
| F2R | -3.237557091 | 0.05074098 |
| LY9 | -3.238372942 | 0.010660904 |
| LINC00643 | -3.24563495 | 0.017581245 |
| LINC00535 | -3.24608335 | 0.031174737 |
| LOC643923 | -3.252102359 | 0.036097133 |
| SLC26A4-AS1 | -3.252382551 | 0.04383421 |
| TSLP | -3.254617695 | 0.044783979 |
| CASP14 | -3.257286217 | 0.026496532 |
| MT1X | -3.262382912 | 0.018833652 |
| GJA3 | -3.273065336 | 0.026066133 |
| HS3ST6 | -3.277869205 | 0.042902153 |
| PTGDR | -3.278258134 | 0.038084453 |
| TRPC7 | -3.278742245 | 0.036783828 |
| AQP6 | -3.280700016 | 0.01875945 |
| IL22RA2 | -3.284310116 | 0.031824724 |
| CPNE4 | -3.284861944 | 0.019442806 |
| RASGEF1C | -3.284905866 | 0.041553401 |
| FAM163A | -3.289568678 | 0.043743041 |
| RNU6-2 | -3.293502856 | 0.040232739 |
| SFRP2 | -3.297763524 | 0.030753271 |
| ALS2CR11 | -3.29787471 | 0.023043758 |
| CENPM | -3.305867603 | 0.019730733 |
| LOC101929696 | -3.312383321 | 0.046242539 |
| HOXA7 | -3.31328892 | 0.046867581 |
| TMEM191A | -3.316341024 | 0.044326773 |
| DOK6 | -3.3224655 | 0.040271618 |
| IL18RAP | -3.324308156 | 0.003848601 |
| TBX10 | -3.330430017 | 0.03451098 |
| FGFBP1 | -3.33554737 | 0.006237984 |
| LINC01125 | -3.33884635 | 0.01904874 |
| LINC00971 | -3.341939401 | 0.029610487 |
| MIP | -3.343366612 | 0.043955584 |
| WDR64 | -3.347022908 | 0.038255642 |
| MYH15 | -3.35192499 | 0.003502457 |
| GAS6-AS2 | -3.359470722 | 0.04626079 |
| IL2RA | -3.365225635 | 0.009785079 |
| ANKRD30B | -3.365799532 | 0.043032425 |
| GAS2 | -3.367092171 | 0.037197717 |
| LOC100507006 | -3.369367616 | 0.023106183 |
| LOC101928443 | -3.374016893 | 0.023332944 |
| CYP11B1 | -3.374637587 | 0.045364632 |
| OTOP2 | -3.375386567 | 0.021823651 |
| PLGLB1 | -3.382303319 | 0.047612284 |
| ANKRD18DP | -3.38475578 | 0.049643139 |
| NLRP14 | -3.38519761 | 0.02435505 |
| LINC01136 | -3.385902217 | 0.012605399 |
| ZP4 | -3.389124137 | 0.049733496 |
| PLGLB2 | -3.393907245 | 0.046141102 |
| ZNF341-AS1 | -3.394128397 | 0.041904844 |
| CYP4F29P | -3.396286266 | 0.016841225 |
| FAM196B | -3.397816807 | 0.001140137 |
| AADACL2 | -3.400725597 | 0.045386489 |
| SYT1 | -3.403272351 | 0.013272292 |
| GDPD4 | -3.405765681 | 0.049385208 |
| ZMAT4 | -3.408357772 | 0.046824891 |
| LRRC37A2 | -3.411464955 | 0.035483121 |
| CSRP3 | -3.417777189 | 0.017485902 |
| DPEP3 | -3.418990875 | 0.034927652 |
| FAM66C | -3.422066517 | 0.010853674 |
| ADAMTS20 | -3.424702273 | 0.040494999 |
| C12orf54 | -3.426968745 | 0.042742539 |
| ST18 | -3.427146239 | 0.013057743 |
| POM121L2 | -3.432071966 | 0.0399527 |
| LOC102723769 | -3.433775767 | 0.049440053 |
| LOC100505474 | -3.435279377 | 0.041146438 |
| GC | -3.4378529 | 0.044811613 |
| GOLGA8S | -3.442605666 | 0.038460384 |
| MTNR1A | -3.443412165 | 0.037307091 |
| C1QTNF7 | -3.446764582 | 0.020726802 |
| CDH17 | -3.44744331 | 0.015690486 |
| FAM182B | -3.447783568 | 0.022164524 |
| HTR3C | -3.448823118 | 0.047349568 |
| PTGER4P2-CDK2AP2P2 | -3.45039158 | 0.031136917 |
| GBP7 | -3.451396816 | 0.009588007 |
| SERPINB4 | -3.453153614 | 0.011452389 |
| TEK | -3.457940433 | 0.022821235 |
| SNORD115-10 | -3.45986171 | 0.049634054 |
| ABCC12 | -3.462227273 | 0.02006188 |
| IGF2BP3 | -3.466368441 | 0.00883025 |
| FGB | -3.468098775 | 0.031649658 |
| SLC4A10 | -3.470084982 | 0.032252387 |
| EYA4-AS1 | -3.472026927 | 0.02961662 |
| ABCG5 | -3.473871087 | 0.028377984 |
| PRR18 | -3.474428251 | 0.014221594 |
| DCDC2C | -3.478668248 | 0.027202501 |
| ASTN2-AS1 | -3.484697963 | 0.022208495 |
| ARRDC5 | -3.488406989 | 0.015102514 |
| OVCH1-AS1 | -3.489749518 | 0.031666929 |
| LINC01353 | -3.491691136 | 0.018528034 |
| ESRG | -3.495586396 | 0.022392566 |
| SERPINB13 | -3.498494152 | 0.007190655 |
| LINC00282 | -3.502551957 | 0.020962603 |
| LINC00222 | -3.503007418 | 0.031744323 |
| NBPF22P | -3.507018929 | 0.047715579 |
| LOC574538 | -3.507426281 | 0.009009532 |
| LOC100128239 | -3.511087964 | 0.021122206 |
| UGT3A1 | -3.51725226 | 0.019295873 |
| LCN1 | -3.517689635 | 0.044762194 |
| CAPNS2 | -3.519205044 | 0.025080495 |
| SYT4 | -3.523029484 | 0.042118409 |
| COL6A4P1 | -3.529372531 | 0.021206948 |
| UBXN10-AS1 | -3.529523476 | 0.016450079 |
| TGM3 | -3.530877825 | 0.004942847 |
| ACTN1-AS1 | -3.53284749 | 0.040142095 |
| MMP20 | -3.532930026 | 0.030133903 |
| LOC399815 | -3.534956925 | 0.025710485 |
| C2orf72 | -3.543890061 | 0.041199133 |
| FBXW12 | -3.544048345 | 0.02717763 |
| CPA4 | -3.547625185 | 0.02752396 |
| DEPDC1 | -3.549511077 | 0.029646398 |
| TPH1 | -3.549790392 | 0.032333534 |
| C5orf66-AS1 | -3.552302084 | 0.028043642 |
| CALY | -3.553608765 | 0.032670308 |
| SLC47A2 | -3.556486665 | 0.023472542 |
| KLRC1 | -3.557499397 | 0.047761446 |
| TEX15 | -3.558731803 | 0.048889812 |
| UCP1 | -3.566911162 | 0.035258211 |
| LINC00880 | -3.568735705 | 0.045353509 |
| FAM71F1 | -3.568894561 | 0.031520558 |
| TCL1B | -3.571980857 | 0.040313491 |
| NAMA | -3.583911841 | 0.026048979 |
| FAM212A | -3.583955986 | 0.029343107 |
| CR1L | -3.587116094 | 0.017758844 |
| TBX2-AS1 | -3.587830884 | 0.03347712 |
| CPO | -3.587944868 | 0.049515078 |
| FOXN3-AS1 | -3.590224901 | 0.010295904 |
| LINC01488 | -3.590427534 | 0.023771487 |
| TBC1D21 | -3.595697014 | 0.035902986 |
| GOLGA8I | -3.597148683 | 0.042071543 |
| CHRNA7 | -3.597519184 | 0.020429174 |
| BDKRB1 | -3.600426358 | 0.036898772 |
| PNPLA5 | -3.607580212 | 0.021706588 |
| C17orf78 | -3.607745258 | 0.044937307 |
| FOXF1 | -3.608390569 | 0.040076083 |
| CYP3A43 | -3.609073045 | 0.024158148 |
| SCN2A | -3.609549679 | 0.016788216 |
| TDRD1 | -3.611580174 | 0.018591455 |
| WTAPP1 | -3.612108806 | 0.002147489 |
| LCT | -3.619678878 | 0.011977177 |
| PDE6G | -3.63181512 | 0.028891811 |
| NLRP8 | -3.63306969 | 0.038089185 |
| NR5A2 | -3.636910732 | 0.035024374 |
| MIR6744 | -3.63788715 | 0.03746648 |
| TGM4 | -3.640361116 | 0.018966938 |
| ACTA2-AS1 | -3.643953813 | 0.013926562 |
| XIST | -3.644921103 | 0.022525118 |
| OLAH | -3.647754544 | 0.034835587 |
| CEACAM22P | -3.652490491 | 0.017812569 |
| PCDH11X | -3.653225457 | 0.038049809 |
| ACSM4 | -3.65323465 | 0.042230409 |
| ADCY10 | -3.653353882 | 0.012033964 |
| DSG1 | -3.654821798 | 0.038323862 |
| MOV10L1 | -3.668575776 | 0.023211223 |
| CACNA2D3-AS1 | -3.669714361 | 0.048554985 |
| GPR128 | -3.671122314 | 0.045237035 |
| PCDHB6 | -3.671927958 | 0.038285481 |
| CLEC4D | -3.676533889 | 0.013984708 |
| KCNA5 | -3.677754473 | 0.02436475 |
| LOC100996634 | -3.682483498 | 0.004665109 |
| RGS7 | -3.683397831 | 0.041463463 |
| PLG | -3.684986249 | 0.01031197 |
| SRPK3 | -3.686021701 | 0.017686987 |
| GUCY1B2 | -3.686303825 | 0.010760817 |
| FAM106A | -3.687084175 | 0.04497321 |
| AKNAD1 | -3.689740646 | 0.031242169 |
| PSG8 | -3.692159795 | 0.042044537 |
| RFPL3S | -3.692451388 | 0.028509901 |
| TCF24 | -3.69450672 | 0.022026123 |
| HTR2A | -3.695238533 | 0.035400099 |
| QRFPR | -3.696456391 | 0.031878535 |
| SLC15A1 | -3.69770171 | 0.013163797 |
| SLC26A7 | -3.698058564 | 0.025450918 |
| ASAP1-IT2 | -3.702011264 | 0.014101839 |
| LOC101929080 | -3.702799554 | 0.046818539 |
| LOC101929181 | -3.703805643 | 0.023724412 |
| KRT17P5 | -3.709244493 | 0.03330291 |
| PCDHB19P | -3.715411384 | 0.027991583 |
| MCHR2 | -3.720090758 | 0.049168035 |
| GPR88 | -3.721916357 | 0.030435942 |
| C10orf99 | -3.722484678 | 0.030146361 |
| SLC22A25 | -3.723062728 | 0.049087663 |
| POSTN | -3.724010964 | 0.021389304 |
| GPR12 | -3.7249308 | 0.01889094 |
| MEDAG | -3.728090765 | 0.018738642 |
| IL1R2 | -3.734479874 | 0.003192305 |
| LOC285768 | -3.735605508 | 0.039499858 |
| LINC01503 | -3.738780696 | 0.005136028 |
| LOC644172 | -3.739244549 | 0.021714952 |
| TMEM187 | -3.745074325 | 0.033782818 |
| TNFSF11 | -3.746115973 | 0.018902021 |
| SLC5A4 | -3.746467713 | 0.027614468 |
| GPR61 | -3.750449942 | 0.02477227 |
| SEMG2 | -3.755730326 | 0.042656832 |
| GAD1 | -3.757847854 | 0.012426927 |
| MCF2 | -3.758341326 | 0.037097124 |
| MPPED1 | -3.76156687 | 0.017707843 |
| OTOP3 | -3.764817469 | 0.035437248 |
| CUZD1 | -3.768792443 | 0.045688129 |
| C10orf91 | -3.77551251 | 0.0240141 |
| CHRNA6 | -3.780851172 | 0.050756489 |
| DDIT4L | -3.782421285 | 0.01454697 |
| MAL | -3.784915804 | 0.03686221 |
| SPDYC | -3.786360226 | 0.020000253 |
| SGCD | -3.78766775 | 0.015895695 |
| LINC00929 | -3.787687959 | 0.045665474 |
| TMEM196 | -3.788655443 | 0.024544721 |
| LINC00682 | -3.796692805 | 0.047660061 |
| LINC01446 | -3.799138473 | 0.050087725 |
| LYZL1 | -3.807307102 | 0.048489016 |
| SERPINA2 | -3.808120555 | 0.041199613 |
| SLC36A3 | -3.808195642 | 0.018879488 |
| INSM2 | -3.808887245 | 0.017236686 |
| USP50 | -3.810434157 | 0.023052028 |
| LINC00264 | -3.813695378 | 0.045610626 |
| CPA5 | -3.818920449 | 0.026128621 |
| APOD | -3.819131413 | 0.000225378 |
| PSG2 | -3.819760956 | 0.038644779 |
| PRAMEF2 | -3.822512239 | 0.04087995 |
| TM6SF2 | -3.833281951 | 0.029197844 |
| GLRA2 | -3.835686618 | 0.034537808 |
| C1orf200 | -3.842804561 | 0.004952883 |
| TUBA3E | -3.844148317 | 0.043029842 |
| XPNPEP2 | -3.847065127 | 0.032703581 |
| NXF3 | -3.854069771 | 0.016020089 |
| GLRA4 | -3.860640628 | 0.030249606 |
| GLRA1 | -3.864060473 | 0.022446368 |
| NXF5 | -3.865014162 | 0.025709401 |
| GRID2 | -3.866117081 | 0.025548432 |
| AKR1C4 | -3.870388239 | 0.036667398 |
| PSG3 | -3.871589982 | 0.038507002 |
| PGBD5 | -3.873261784 | 0.006790424 |
| GOLGA8F | -3.874786656 | 0.032653032 |
| ZP3 | -3.878635683 | 0.021800442 |
| LOC101928940 | -3.880191222 | 0.047314501 |
| GOLGA8G | -3.880646609 | 0.03177848 |
| GABRA5 | -3.881359615 | 0.032661971 |
| FIBIN | -3.887180305 | 0.026560661 |
| LOC101927619 | -3.89514142 | 0.020568737 |
| ANAPC1P1 | -3.89576124 | 0.018895848 |
| IL1RL1 | -3.901686317 | 0.006523593 |
| SERPINB9P1 | -3.905130959 | 0.002572065 |
| PLP1 | -3.906043415 | 0.017631532 |
| LINC00595 | -3.907004436 | 0.037530846 |
| OR51E1 | -3.913251564 | 0.035070315 |
| AQP8 | -3.914230158 | 0.048256883 |
| SFRP4 | -3.915443081 | 0.014062055 |
| ADORA2B | -3.915690743 | 0.000856045 |
| AGT | -3.918100472 | 0.021948378 |
| ENPP6 | -3.923075452 | 0.02489985 |
| BPIFA4P | -3.926824602 | 0.015687301 |
| LOC101929723 | -3.928421644 | 0.036486186 |
| SLC10A6 | -3.928573022 | 0.014997243 |
| PROZ | -3.932230904 | 0.019155597 |
| UNC5B-AS1 | -3.932339616 | 0.024860625 |
| PCDHB17 | -3.932370726 | 0.034601775 |
| ADIPOQ | -3.934640635 | 0.016846393 |
| ZNF812 | -3.942836289 | 0.00235307 |
| FRMD7 | -3.950716875 | 0.015996668 |
| EBF3 | -3.952632595 | 0.005228979 |
| CELSR3-AS1 | -3.954969787 | 0.025027482 |
| RSU1P2 | -3.963144812 | 0.039964051 |
| LOC391322 | -3.971254321 | 0.022375641 |
| CNTNAP4 | -3.97198271 | 0.011080725 |
| LOC101927166 | -3.977197754 | 0.040820746 |
| LOC440600 | -3.978256842 | 0.022156577 |
| ACY3 | -3.979587212 | 0.018245593 |
| SH3GL3 | -3.991875139 | 0.007760716 |
| OR7E5P | -3.994295761 | 0.044336589 |
| LINC01516 | -3.996630599 | 0.046063711 |
| CD1B | -3.997328934 | 0.021268126 |
| TBC1D29 | -3.999463602 | 0.012187421 |
| LINC01449 | -3.999993192 | 0.046428308 |
| LOC101927136 | -4.000645432 | 0.017360325 |
| LINC00856 | -4.011204495 | 0.045989103 |
| CREG2 | -4.015561129 | 0.036205121 |
| ALDOB | -4.015743637 | 0.011475372 |
| IL36B | -4.018022346 | 0.030036084 |
| C22orf42 | -4.024769442 | 0.044659736 |
| AVP | -4.026841481 | 0.042691144 |
| HAO2 | -4.037382866 | 0.033672712 |
| BFSP2 | -4.042645237 | 0.021590157 |
| SPATA31C1 | -4.042692699 | 0.012031949 |
| ZBTB46-AS1 | -4.042737131 | 0.015939118 |
| LOC101930452 | -4.043168568 | 0.01004806 |
| LOC389641 | -4.045812015 | 0.007624749 |
| CA5A | -4.049438177 | 0.031379689 |
| LINC00536 | -4.050790539 | 0.025056325 |
| FAM212B-AS1 | -4.054996339 | 0.025506367 |
| LEFTY2 | -4.05503737 | 0.019009889 |
| DYTN | -4.059583146 | 0.029373951 |
| LINC00670 | -4.061379412 | 0.028831371 |
| ULBP1 | -4.074727028 | 0.026661345 |
| LOC101929690 | -4.087166235 | 0.023986844 |
| A1CF | -4.093490278 | 0.013715464 |
| GOLGA6L7P | -4.099776968 | 0.013317388 |
| LRIT2 | -4.102231871 | 0.04350226 |
| SNX31 | -4.10531497 | 0.031578726 |
| GOLGA6A | -4.105572894 | 0.020516927 |
| MYT1L-AS1 | -4.106295708 | 0.015123719 |
| WNT7A | -4.123314192 | 0.009686439 |
| KCNK17 | -4.124326504 | 0.013621251 |
| WFDC1 | -4.128989026 | 0.016605785 |
| TBX5-AS1 | -4.130050466 | 0.010448685 |
| TMPRSS15 | -4.132918986 | 0.019022803 |
| LOC101928107 | -4.136070998 | 0.028913087 |
| RFX8 | -4.145346677 | 0.014559989 |
| ADH1A | -4.148343827 | 0.012137996 |
| HRH2 | -4.156756141 | 0.00199442 |
| SERPINA6 | -4.157425399 | 0.01394338 |
| RAET1L | -4.160760319 | 0.031774056 |
| BRDT | -4.161032302 | 0.015731916 |
| C8G | -4.162180128 | 0.014725582 |
| CACNG1 | -4.162241682 | 0.012538557 |
| IRGM | -4.163576745 | 0.018473118 |
| SLCO5A1 | -4.167047379 | 0.006589696 |
| LINC01101 | -4.167421349 | 0.020559035 |
| LOC440704 | -4.171132834 | 0.033861474 |
| LOC101928322 | -4.174243201 | 0.033914474 |
| GOLGA6C | -4.175672737 | 0.03886995 |
| SLC7A10 | -4.17728475 | 0.011891328 |
| LINC00200 | -4.177397566 | 0.021676042 |
| HIGD1B | -4.180586331 | 0.026486459 |
| TRIM42 | -4.185739057 | 0.039520355 |
| SERPINA11 | -4.186473533 | 0.022795198 |
| ADAMTS19 | -4.187521879 | 0.008731482 |
| AKR1D1 | -4.191706427 | 0.023428875 |
| EFTUD1P1 | -4.196797455 | 0.003172456 |
| PRSS33 | -4.206644177 | 0.022642922 |
| CYP26C1 | -4.214256828 | 0.028281409 |
| NOL4 | -4.214436132 | 0.030709745 |
| SLC5A12 | -4.214892116 | 0.006160381 |
| TMEM74 | -4.215984806 | 0.029383957 |
| GRK1 | -4.219057171 | 0.008996596 |
| GOLGA8J | -4.219778 | 0.021257985 |
| SLC25A47 | -4.223813145 | 0.016800025 |
| LOC101927284 | -4.229446569 | 0.030867739 |
| KCTD16 | -4.235783969 | 0.010451995 |
| LOC101928894 | -4.236217314 | 0.023128444 |
| TBC1D3P2 | -4.2418396 | 0.038699168 |
| LRRC10 | -4.251114719 | 0.028941848 |
| CCDC63 | -4.265871587 | 0.022809875 |
| LINC00964 | -4.274257687 | 0.013596211 |
| CLEC18B | -4.275242045 | 0.002843681 |
| RESP18 | -4.275818695 | 0.032136404 |
| RPTN | -4.283285658 | 0.013484478 |
| UBQLNL | -4.283614687 | 0.015026987 |
| UPK2 | -4.290622472 | 0.008803003 |
| SLC9B2 | -4.291187998 | 0.005225007 |
| PWRN2 | -4.292956868 | 0.018670233 |
| RGAG1 | -4.302401543 | 0.021207668 |
| CTNND2 | -4.307823792 | 0.002814611 |
| CHEK2P2 | -4.311257635 | 0.049645492 |
| SRPX | -4.326310326 | 0.019593893 |
| PSG1 | -4.330676823 | 0.015008986 |
| CRYBA2 | -4.331675683 | 0.02020354 |
| PDE6C | -4.332842597 | 0.015907562 |
| CDH13 | -4.345157106 | 0.002035173 |
| PPAPDC3 | -4.347534079 | 0.005552216 |
| LEFTY1 | -4.353843461 | 0.011163248 |
| UPB1 | -4.363865344 | 0.012268424 |
| GUCA1A | -4.364066795 | 0.018219576 |
| IFNL4 | -4.366186087 | 0.016429978 |
| LOC100133050 | -4.372353442 | 0.009637383 |
| LOC339298 | -4.38447485 | 0.011144637 |
| AADACL4 | -4.38450474 | 0.030523221 |
| FAM230C | -4.389951116 | 0.025200243 |
| C1orf140 | -4.394423267 | 0.017896532 |
| DSG3 | -4.411967199 | 0.002820326 |
| CCDC144NL | -4.41252865 | 0.0228584 |
| MYL10 | -4.420727674 | 0.019102249 |
| MYO1H | -4.437163644 | 0.004743106 |
| GCM1 | -4.43842977 | 0.009427688 |
| LOC285819 | -4.438447605 | 0.006738465 |
| VSTM2B | -4.447796784 | 0.016444611 |
| LAMB4 | -4.449659106 | 0.002529289 |
| PFKFB1 | -4.451349256 | 0.004592384 |
| SI | -4.458949787 | 0.013312291 |
| NXF4 | -4.46174919 | 0.015227866 |
| RIMBP3 | -4.462200128 | 0.00481004 |
| KLRC3 | -4.468792342 | 0.009794523 |
| DIO3OS | -4.470996463 | 0.002720434 |
| ANTXRLP1 | -4.478699613 | 0.011673629 |
| KIAA1024 | -4.484913217 | 0.012304298 |
| KIR2DL3 | -4.48669556 | 0.009737019 |
| MTNR1B | -4.488823031 | 0.00962605 |
| MIR143HG | -4.490507318 | 0.004369752 |
| LOC100129055 | -4.497154222 | 0.011189636 |
| TMEM249 | -4.511115812 | 0.010839529 |
| UPK3A | -4.537388368 | 0.018121613 |
| LBP | -4.553842975 | 0.006107807 |
| FOXD4 | -4.559737028 | 0.014521827 |
| PRAMEF1 | -4.561822104 | 0.018868473 |
| TENM1 | -4.575509252 | 0.000666371 |
| CES5A | -4.576156851 | 0.00453897 |
| VIT | -4.581799297 | 0.008857222 |
| LINC01005 | -4.592201113 | 0.012659054 |
| ULBP2 | -4.593205723 | 0.011079904 |
| CLEC17A | -4.594734097 | 0.00346185 |
| RAB39B | -4.600425865 | 0.005664018 |
| AOX2P | -4.603930202 | 0.0085681 |
| MIR326 | -4.612917431 | 0.03511048 |
| HEATR9 | -4.624602995 | 0.002076292 |
| SLC22A14 | -4.627658816 | 0.004939049 |
| FLJ42102 | -4.640913902 | 0.00595312 |
| LOC149950 | -4.647664927 | 0.019801171 |
| RPL3L | -4.649651488 | 0.014851981 |
| LINC01447 | -4.653446465 | 0.020252215 |
| FSHR | -4.699843356 | 0.00445788 |
| AWAT2 | -4.700114184 | 0.005516606 |
| SPATA31D1 | -4.701613593 | 0.007513088 |
| XKR4 | -4.736866959 | 0.005334331 |
| CNIH3 | -4.738716871 | 0.000430525 |
| LINC01163 | -4.74151541 | 0.004458491 |
| CIDEC | -4.755356767 | 0.003309042 |
| LOC101927683 | -4.760283733 | 0.027158745 |
| GATA5 | -4.805216781 | 0.002642327 |
| TNFRSF8 | -4.807789507 | 0.00062524 |
| DMBT1P1 | -4.813833809 | 0.001730735 |
| LRP1B | -4.822829299 | 0.001290815 |
| EPO | -4.825366812 | 0.004570226 |
| LINC-ROR | -4.877989397 | 0.006777825 |
| MIR6723 | -4.91051052 | 0.000418052 |
| SLC6A10P | -4.930223327 | 0.007264547 |
| LOC388553 | -5.007718393 | 0.004081783 |
| LINC01134 | -5.0243249 | 0.001561852 |
| SAG | -5.124577091 | 0.000883605 |
| WDR88 | -5.165045354 | 0.001459083 |
| TSG1 | -5.216493307 | 0.005385614 |
| C14orf180 | -5.307617546 | 0.002188943 |
| LPA | -5.332371477 | 0.000298871 |
| WNT2 | -5.389055009 | 0.003491761 |
| LINC00521 | -5.469696657 | 0.00193488 |
| ST8SIA1 | -5.705136318 | 0.000215501 |

Table S3: Differentially expressed genes in isolated AEC2 cells. List of all differentially expressed genes in AEC2 cells of patients with IPF with history of moderate COVID compared to COVID-ARDS group. *p < 0.05

| **Gene Symbol** | **log2FoldChange** | **p-value** |
| --- | --- | --- |
| ST8SIA1 | 5.807975217 | 0.000775324 |
| LINC00521 | 5.641291228 | 0.003313892 |
| C14orf180 | 5.056211843 | 0.010086084 |
| LRP1B | 4.972302813 | 0.001884018 |
| SAG | 4.892274164 | 0.010582302 |
| CNIH3 | 4.886376561 | 0.001566617 |
| LOC388553 | 4.878664742 | 0.020198303 |
| WDR88 | 4.867470192 | 0.015622231 |
| LPA | 4.857371804 | 0.004910749 |
| KIR2DL5A | 4.74715789 | 0.050275261 |
| KIR2DL5B | 4.74715789 | 0.050275261 |
| GATA5 | 4.733866027 | 0.007908693 |
| UPK3A | 4.686943645 | 0.03231611 |
| RPL3L | 4.673639145 | 0.028816183 |
| DMBT1P1 | 4.673182489 | 0.009135678 |
| VSTM2B | 4.666935825 | 0.025133566 |
| LINC01163 | 4.661507115 | 0.018036414 |
| RIMBP3 | 4.644626657 | 0.007334754 |
| EPO | 4.589693781 | 0.031071913 |
| LEFTY1 | 4.582809277 | 0.01538023 |
| GUCA1A | 4.556671714 | 0.032559118 |
| XKR4 | 4.547313349 | 0.025094413 |
| SI | 4.538341983 | 0.034552423 |
| LINC01134 | 4.533338993 | 0.019257483 |
| SLC22A14 | 4.504634791 | 0.024015466 |
| GOLGA8J | 4.498216496 | 0.033462506 |
| RPTN | 4.48104423 | 0.031155813 |
| LINC00964 | 4.469777695 | 0.031501458 |
| DIO3OS | 4.4579403 | 0.009101227 |
| GOLGA6A | 4.457086525 | 0.021104504 |
| LBP | 4.446210975 | 0.019412645 |
| FLJ42102 | 4.409823998 | 0.03809796 |
| HEATR9 | 4.399978882 | 0.013014212 |
| TMEM249 | 4.384733851 | 0.039616207 |
| RFX8 | 4.375659445 | 0.024081995 |
| RAB39B | 4.37166877 | 0.029842936 |
| PWRN2 | 4.323520291 | 0.050900798 |
| MYO1H | 4.322557591 | 0.019451629 |
| KIAA1024 | 4.31106498 | 0.04727956 |
| EFTUD1P1 | 4.291571383 | 0.025081932 |
| TENM1 | 4.288042376 | 0.010284419 |
| BFSP2 | 4.275216212 | 0.034985899 |
| LOC285819 | 4.269219677 | 0.037606989 |
| AOX2P | 4.265717932 | 0.046741574 |
| VIT | 4.25141735 | 0.048508285 |
| PDE6C | 4.250261979 | 0.049101081 |
| KCTD16 | 4.241772271 | 0.028317713 |
| NXF4 | 4.231593488 | 0.044070639 |
| SLC9B2 | 4.221528606 | 0.030770993 |
| SLC7A10 | 4.208093156 | 0.028151335 |
| HRH2 | 4.19818234 | 0.009302687 |
| CES5A | 4.188404326 | 0.032150177 |
| CACNG1 | 4.183640426 | 0.027864677 |
| ENPP6 | 4.162185925 | 0.037760388 |
| SERPINA6 | 4.147800112 | 0.049414095 |
| A1CF | 4.132907398 | 0.036065304 |
| LOC100996634 | 4.131435205 | 0.003933554 |
| CDH13 | 4.114215652 | 0.014577721 |
| TNFRSF8 | 4.111785511 | 0.012729207 |
| CD1B | 4.104585539 | 0.049748102 |
| SLC5A12 | 4.097707895 | 0.026310634 |
| LAMB4 | 4.095795868 | 0.018576934 |
| KCNK17 | 4.09166042 | 0.038093735 |
| PPAPDC3 | 4.078535648 | 0.032601547 |
| SLCO5A1 | 4.065916853 | 0.020948213 |
| GRK1 | 4.044918048 | 0.030069746 |
| RGS7 | 4.014673364 | 0.049609262 |
| INSM2 | 3.999999172 | 0.035666816 |
| ZP3 | 3.970852884 | 0.03891531 |
| PLG | 3.965176513 | 0.012411308 |
| TAP2 | 3.943071999 | 0.024573125 |
| SFRP4 | 3.94010078 | 0.040754976 |
| OTOP3 | 3.937749564 | 0.044325258 |
| NLRP8 | 3.914795436 | 0.043976 |
| CTNND2 | 3.899067823 | 0.030016022 |
| GAD1 | 3.893023256 | 0.020559019 |
| BPIFA4P | 3.877965938 | 0.046911807 |
| SH3GL3 | 3.860152172 | 0.02859601 |
| SLC26A7 | 3.850070756 | 0.044611908 |
| EBF3 | 3.843777995 | 0.016510876 |
| GPR61 | 3.840417142 | 0.044805129 |
| ASAP1-IT2 | 3.822257714 | 0.045687553 |
| CDH17 | 3.819821927 | 0.012504037 |
| UBXN10-AS1 | 3.806682799 | 0.028890268 |
| SLC26A4-AS1 | 3.789743366 | 0.035570188 |
| PRR18 | 3.773003404 | 0.016982456 |
| MOV10L1 | 3.767754321 | 0.032669272 |
| ACTA2-AS1 | 3.756202328 | 0.034979201 |
| SFRP2 | 3.749446045 | 0.023516546 |
| GOLGA8S | 3.736272036 | 0.041748577 |
| DSG3 | 3.732594582 | 0.039668428 |
| SGCD | 3.730796547 | 0.033334999 |
| IL1RL1 | 3.646905706 | 0.049514679 |
| CLEC18B | 3.635009283 | 0.032274885 |
| ST18 | 3.619443224 | 0.021302302 |
| LINC01125 | 3.602855166 | 0.027656129 |
| C1QTNF7 | 3.593963349 | 0.049656929 |
| GBP7 | 3.587489704 | 0.036034919 |
| SLC22A16 | 3.582990919 | 0.030295118 |
| FCGR2B | 3.558360694 | 0.012355077 |
| IGF2BP3 | 3.556906238 | 0.032436779 |
| ADORA2B | 3.549479327 | 0.032514556 |
| LRRC63 | 3.545353107 | 0.035527628 |
| SLC15A1 | 3.543393884 | 0.04997972 |
| PTX3 | 3.541011048 | 0.038551314 |
| CENPM | 3.539535491 | 0.031399177 |
| NLRP14 | 3.531601813 | 0.038162228 |
| GUCY1B2 | 3.530389157 | 0.041398472 |
| LINC01136 | 3.51658309 | 0.036361623 |
| LINC00643 | 3.478971638 | 0.041596623 |
| TDRD1 | 3.453216169 | 0.050278521 |
| SERPINB4 | 3.44472405 | 0.029989644 |
| LCT | 3.441909782 | 0.039013242 |
| CD244 | 3.436464723 | 0.023467283 |
| STAT4 | 3.363784959 | 0.022188313 |
| LOC574538 | 3.362630642 | 0.034454146 |
| LAMA2 | 3.346285157 | 0.000944643 |
| ABCB1 | 3.339484236 | 0.03304367 |
| ENPP3 | 3.333114085 | 0.033890547 |
| LY9 | 3.325661043 | 0.015340197 |
| TGM3 | 3.325106262 | 0.025211286 |
| GNN | 3.296061981 | 0.014786893 |
| TXNDC8 | 3.294654743 | 0.021522894 |
| RHOH | 3.252456472 | 0.011099613 |
| CPNE4 | 3.247656276 | 0.049926341 |
| CD207 | 3.188984841 | 0.007733183 |
| MYH15 | 3.181748869 | 0.014646705 |
| CCDC13-AS1 | 3.168324553 | 0.032566576 |
| SLC44A5 | 3.164203294 | 0.030257453 |
| CD226 | 3.157097109 | 0.035860512 |
| MMP12 | 3.142487388 | 0.043041566 |
| CHDC2 | 3.114157659 | 0.039358992 |
| CD48 | 3.110626816 | 0.035930216 |
| TRIP13 | 3.104515212 | 0.023445863 |
| BEST4 | 3.078157391 | 0.022069922 |
| ABCA6 | 3.078116321 | 0.024405472 |
| FAM196B | 3.076042476 | 0.033941366 |
| BAALC | 3.069199689 | 0.048436285 |
| APOD | 3.068655302 | 0.020861203 |
| TGFBR3L | 3.044704823 | 0.044624858 |
| FCGR2C | 3.035629358 | 0.011199661 |
| SPAG4 | 3.02194498 | 0.040969235 |
| ACTA2 | 3.015353186 | 0.050884503 |
| NRCAM | 2.994486208 | 0.027643734 |
| FGFBP1 | 2.977455576 | 0.040598102 |
| CD1C | 2.962085311 | 0.021985622 |
| GOLGA8H | 2.955198462 | 0.024059562 |
| EFHC2 | 2.91025658 | 0.014408446 |
| FSIP2 | 2.891004064 | 0.011241039 |
| PTGIS | 2.880975257 | 0.046474976 |
| NLRP3 | 2.877085159 | 0.002858284 |
| CHADL | 2.846422897 | 0.022724087 |
| EGF | 2.838415281 | 0.050007427 |
| APOBEC3G | 2.837036915 | 0.044812481 |
| MCAM | 2.825487205 | 0.034983536 |
| ARL6 | 2.796612058 | 0.03571772 |
| JAKMIP1 | 2.766124992 | 0.049617726 |
| LOC101929653 | 2.755956294 | 0.037771507 |
| PKD1L1 | 2.755245518 | 0.04682876 |
| IPCEF1 | 2.753083875 | 0.021978622 |
| CD96 | 2.746100129 | 0.046626377 |
| ATP8B4 | 2.735290716 | 0.003991466 |
| TCTEX1D1 | 2.730746286 | 0.048591555 |
| CTSK | 2.726958709 | 0.011425927 |
| SCARF2 | 2.708725971 | 0.019818036 |
| GRM7 | 2.699145492 | 0.02615462 |
| C1orf110 | 2.696565663 | 0.019058615 |
| LMNTD1 | 2.673436101 | 0.017150948 |
| TGFBR3 | 2.652330883 | 0.011610867 |
| FDPSP2 | 2.635755934 | 0.031449386 |
| DCLK2 | 2.63195841 | 0.049803704 |
| CAPSL | 2.626380135 | 0.027174606 |
| FCGR1A | 2.611810705 | 0.040544328 |
| SLC26A4 | 2.609054545 | 0.037448867 |
| ALDH3A1 | 2.594603518 | 0.008190318 |
| SLC9A3 | 2.585672538 | 0.013050057 |
| ZAP70 | 2.580139929 | 0.022420241 |
| CHIT1 | 2.569029533 | 0.004018979 |
| FBXL2 | 2.556512581 | 0.046530065 |
| LINC00202-1 | 2.549308569 | 0.044343011 |
| SYT8 | 2.534020649 | 0.013707672 |
| PDZD4 | 2.524057535 | 0.013609795 |
| SNCA | 2.520856998 | 0.034369355 |
| ITGA4 | 2.517466959 | 0.004592248 |
| ADAMTS14 | 2.515782883 | 0.01798431 |
| FRMPD2 | 2.509830161 | 0.011138975 |
| COL3A1 | 2.488428288 | 0.037023064 |
| DUSP2 | 2.481647603 | 0.035140513 |
| CYP24A1 | 2.480656303 | 0.034248069 |
| MROH9 | 2.473455878 | 0.022781453 |
| DCDC5 | 2.449968195 | 0.013958335 |
| EFHC1 | 2.446699663 | 0.010123601 |
| DNAH3 | 2.440194482 | 0.004968424 |
| CXorf22 | 2.43759145 | 0.028606709 |
| ADAMTS15 | 2.437133302 | 0.039660861 |
| SGSM1 | 2.41047112 | 0.037732083 |
| ANKUB1 | 2.406588349 | 0.048162781 |
| BCO2 | 2.399570539 | 0.017506305 |
| LOC100288152 | 2.398271733 | 0.032087209 |
| TRPC6 | 2.349376489 | 0.039001478 |
| TMPRSS4 | 2.341176642 | 0.010140918 |
| E2F8 | 2.337101791 | 0.049619765 |
| RSPH9 | 2.332868635 | 0.033573368 |
| TRPM2 | 2.296080943 | 0.01572093 |
| KIAA0319 | 2.27774552 | 0.007570879 |
| NLRC3 | 2.27488176 | 0.016989774 |
| VCAN | 2.272001427 | 0.012748517 |
| C11orf70 | 2.271248045 | 0.037952125 |
| LEKR1 | 2.262851609 | 0.049340561 |
| SLC22A4 | 2.241649446 | 0.017504543 |
| EML6 | 2.220049994 | 0.006996377 |
| ARHGAP42 | 2.198206134 | 0.02225523 |
| RAB36 | 2.190767855 | 0.02528042 |
| TEKT4 | 2.186564995 | 0.034214863 |
| SYNE3 | 2.145490817 | 0.011971733 |
| ADAM19 | 2.139773662 | 0.00490625 |
| CYP1B1 | 2.139254997 | 0.043939609 |
| PTPRN2 | 2.135996205 | 0.033910541 |
| TMEM231 | 2.133862838 | 0.035153304 |
| ICA1L | 2.098867469 | 0.016607425 |
| AK8 | 2.071092009 | 0.044257203 |
| FAM118A | 2.058750588 | 0.050118598 |
| CDHR4 | 2.058290841 | 0.03273713 |
| FCN1 | 2.053829714 | 0.019398255 |
| CCDC17 | 2.047295641 | 0.044118603 |
| NELL2 | 2.038162196 | 0.018468692 |
| C6orf165 | 2.03037829 | 0.047682962 |
| ABCA5 | 2.0116525 | 0.03240477 |
| CCDC30 | 2.007137127 | 0.038278862 |
| DNAH6 | 1.998264127 | 0.017804923 |
| LOC200772 | 1.996756492 | 0.031941099 |
| KCNN4 | 1.990061442 | 0.038605741 |
| DNAH9 | 1.988596245 | 0.017077879 |
| DYNC2H1 | 1.987280653 | 0.020241197 |
| DNAH12 | 1.982510041 | 0.033529586 |
| BEST1 | 1.976964846 | 0.035657406 |
| CYTIP | 1.963001914 | 0.039223221 |
| CDS1 | 1.95883077 | 0.035770886 |
| FAM179A | 1.943692064 | 0.012234186 |
| WDR16 | 1.92355604 | 0.03118285 |
| LMLN | 1.916382049 | 0.044730016 |
| AKAP6 | 1.908267271 | 0.017769379 |
| HDAC9 | 1.906956047 | 0.038363668 |
| WDR54 | 1.905646568 | 0.028831728 |
| WDR19 | 1.878864932 | 0.047972108 |
| IL5RA | 1.876964803 | 0.032475653 |
| ICAM2 | 1.875691382 | 0.038405228 |
| BCL2 | 1.867361831 | 0.031808705 |
| ZBBX | 1.853752201 | 0.048959711 |
| DNAH7 | 1.842762171 | 0.035305094 |
| VOPP1 | 1.825887642 | 0.04297743 |
| FAM198B | 1.819713342 | 0.042570704 |
| DLEC1 | 1.809481291 | 0.019408726 |
| GPR110 | 1.791619567 | 0.033317542 |
| TTC21A | 1.787843474 | 0.030740954 |
| LRRC48 | 1.781933683 | 0.03649334 |
| RP1 | 1.773617454 | 0.038806754 |
| CFAP61 | 1.770479227 | 0.041093469 |
| DNAH11 | 1.768935266 | 0.024294331 |
| LILRB4 | 1.763645485 | 0.032250708 |
| CR1 | 1.758391585 | 0.025936281 |
| WDR90 | 1.756912 | 0.048564615 |
| TTC18 | 1.756815949 | 0.034859199 |
| PRKX | 1.749601374 | 0.049032113 |
| NWD1 | 1.740372536 | 0.029268104 |
| CCDC13 | 1.735844664 | 0.024891386 |
| PRKCB | 1.71885945 | 0.020930625 |
| SPEF2 | 1.713808936 | 0.044938672 |
| LOC101927267 | 1.712949108 | 0.042744386 |
| C20orf96 | 1.694768311 | 0.048832858 |
| BAIAP3 | 1.68934645 | 0.029071951 |
| VWA3A | 1.675946035 | 0.032382609 |
| ADAM12 | 1.671332007 | 0.041259692 |
| DGKH | 1.664732255 | 0.019178514 |
| AOAH | 1.645121419 | 0.039863189 |
| DQX1 | 1.619471446 | 0.050878048 |
| ITGB8 | 1.605759876 | 0.041275472 |
| EMR2 | 1.601356751 | 0.037957796 |
| AGBL2 | 1.595193925 | 0.048812513 |
| PKD1 | 1.583913195 | 0.028789259 |
| IL16 | 1.583772822 | 0.018481534 |
| DNAH1 | 1.557953606 | 0.025671027 |
| ENTPD1 | 1.538730358 | 0.023245794 |
| PTPRT | 1.522850688 | 0.047142294 |
| C21orf58 | 1.477138789 | 0.050466553 |
| AGPAT4 | 1.459301478 | 0.049679725 |
| FMNL1 | 1.44466138 | 0.024178308 |
| PRUNE2 | 1.392975738 | 0.04837937 |
| PHF23 | -1.235321129 | 0.046078502 |
| GLS | -1.243529466 | 0.04311882 |
| DMBT1 | -1.33163546 | 0.034701974 |
| ACADSB | -1.356372289 | 0.031850121 |
| PBXIP1 | -1.363353283 | 0.041267334 |
| NAB2 | -1.367934708 | 0.04320137 |
| DBN1 | -1.409298559 | 0.025992333 |
| TCP11 | -1.410499071 | 0.046214496 |
| HDDC2 | -1.422548322 | 0.049601992 |
| AK1 | -1.42809032 | 0.02946889 |
| PXMP4 | -1.449063555 | 0.03843126 |
| MTA2 | -1.45217045 | 0.026791622 |
| FRA10AC1 | -1.45246297 | 0.046670473 |
| FADS2 | -1.455565143 | 0.026958224 |
| LOC729867 | -1.467892169 | 0.045522516 |
| PRRT2 | -1.487890572 | 0.024722278 |
| SLC25A10 | -1.504990471 | 0.024373296 |
| NFIX | -1.528384628 | 0.011868569 |
| FBXW4 | -1.543274644 | 0.020331304 |
| RSBN1L | -1.543771697 | 0.038923532 |
| SPSB2 | -1.550497658 | 0.047978199 |
| ZBTB47 | -1.554678177 | 0.033294143 |
| SLC15A3 | -1.557936033 | 0.043700341 |
| CREB3L1 | -1.559579708 | 0.025245971 |
| LOC100652999 | -1.569038609 | 0.020556398 |
| SERPING1 | -1.570452688 | 0.045684557 |
| SCD | -1.572143907 | 0.044371169 |
| RAB21 | -1.575137896 | 0.035983884 |
| RAB5C | -1.580826313 | 0.034849472 |
| IL11RA | -1.580894887 | 0.031660258 |
| BCAM | -1.591874113 | 0.028296852 |
| NR1H2 | -1.599301975 | 0.015339406 |
| LYRM1 | -1.601074922 | 0.021233403 |
| SMARCA5-AS1 | -1.608429949 | 0.039164334 |
| FBL | -1.619811789 | 0.031124342 |
| SNRPD2 | -1.630442272 | 0.03535009 |
| MUC1 | -1.643568249 | 0.027484977 |
| NDNF | -1.657442366 | 0.036080799 |
| ZNF503 | -1.681380488 | 0.034899546 |
| PPP1R1B | -1.707926248 | 0.018934135 |
| LAMTOR1 | -1.720160003 | 0.021607057 |
| GALNT5 | -1.728035897 | 0.04009796 |
| C19orf68 | -1.729969742 | 0.004420582 |
| PTRF | -1.731333395 | 0.018964546 |
| RHOBTB2 | -1.753035911 | 0.017172153 |
| CADM1 | -1.764264669 | 0.029950558 |
| CDC42EP1 | -1.767927006 | 0.020522597 |
| LINC01354 | -1.768172968 | 0.040743111 |
| KCNC3 | -1.770469279 | 0.01760993 |
| INSIG2 | -1.775666571 | 0.016118041 |
| HAGLR | -1.805082775 | 0.036194943 |
| NPNT | -1.814508374 | 0.022921477 |
| B3GALT6 | -1.816130214 | 0.025892598 |
| ROS1 | -1.821863312 | 0.048700041 |
| NDUFA12 | -1.82220811 | 0.047581386 |
| GTF2H2C_2 | -1.827313166 | 0.04749457 |
| THEG5 | -1.864713305 | 0.012426979 |
| GTF2H2C | -1.870892921 | 0.041326598 |
| SZRD1 | -1.881624663 | 0.005087823 |
| NEDD8-MDP1 | -1.88196526 | 0.034842657 |
| GFER | -1.888234639 | 0.022037824 |
| NHLH1 | -1.89184993 | 0.033754933 |
| ELOVL5 | -1.900823697 | 0.008562308 |
| COA6 | -1.903445096 | 0.036941015 |
| CLDN18 | -1.908823677 | 0.033355998 |
| FBXL19 | -1.909837981 | 0.033009456 |
| APTR | -1.923270408 | 0.011741228 |
| JUND | -1.937737806 | 0.010406122 |
| C1R | -1.942525155 | 0.014578654 |
| NDUFA6 | -1.944777256 | 0.012568067 |
| NDUFA4 | -1.94703046 | 0.011739053 |
| INHBA | -1.95236797 | 0.050510898 |
| NUPR1 | -1.978480305 | 0.033865078 |
| SLC39A8 | -1.984585701 | 0.003221134 |
| HDAC6 | -2.00301822 | 0.005478937 |
| NFATC4 | -2.038398993 | 0.001189418 |
| PALM3 | -2.040623449 | 0.032521586 |
| FHDC1 | -2.052137823 | 0.000685133 |
| NAP1L5 | -2.085381931 | 0.019735023 |
| PARM1 | -2.094836824 | 0.000891068 |
| KIAA0408 | -2.095191867 | 0.02027043 |
| LRRK2 | -2.113849169 | 0.00202354 |
| SSTR5-AS1 | -2.127431574 | 0.001089912 |
| CA11 | -2.140113419 | 0.01208428 |
| SFTA3 | -2.174481786 | 0.029363549 |
| C1QB | -2.188205643 | 0.017106793 |
| SFTA1P | -2.202924692 | 0.020597755 |
| MEX3A | -2.217187491 | 0.006758803 |
| LPCAT1 | -2.228059239 | 0.001142245 |
| ALPL | -2.231730147 | 0.002654597 |
| UBB | -2.234433977 | 0.031852649 |
| CACNA2D2 | -2.235533743 | 0.016180352 |
| NRGN | -2.291137931 | 0.008348468 |
| SFTPB | -2.322920861 | 0.020837548 |
| LSM4 | -2.32513311 | 0.001642755 |
| SNORD33 | -2.3942762 | 0.010790483 |
| IL21-AS1 | -2.396747919 | 0.002247187 |
| KCNJ15 | -2.405602924 | 0.003036046 |
| SCN5A | -2.411003999 | 0.011616206 |
| GJA1 | -2.422208573 | 0.009167448 |
| STXBP5-AS1 | -2.43111052 | 0.020406928 |
| RND1 | -2.466283381 | 0.010362496 |
| CGNL1 | -2.467408002 | 0.005093287 |
| AQP1 | -2.496205526 | 4.43766E-05 |
| S100A14 | -2.542804305 | 0.000259093 |
| PGC | -2.612544256 | 0.02864113 |
| ABCA3 | -2.617356395 | 0.005240396 |
| HOXC8 | -2.653243322 | 0.049391504 |
| LOC101927587 | -2.715250405 | 0.03927812 |
| CACNG7 | -2.720666816 | 0.039326664 |
| MFI2-AS1 | -2.727859062 | 0.026474525 |
| LAMP3 | -2.728432822 | 0.000398918 |
| BEX2 | -2.763154779 | 0.000486686 |
| SFRP5 | -2.765566698 | 0.004200723 |
| SNORD95 | -2.801439689 | 0.000873388 |
| SNORD57 | -2.815408248 | 0.000698049 |
| PTPRD-AS1 | -2.876547221 | 0.036676581 |
| SNORD41 | -3.073070421 | 0.000485164 |
| CHAC2 | -3.090065833 | 0.009344145 |
| WDFY3-AS2 | -3.138654476 | 0.005592995 |
| TCEAL2 | -3.176766651 | 0.005131942 |
| ACR | -3.25711562 | 0.011103026 |
| MIR320C1 | -3.279682008 | 0.030745544 |
| MIR2276 | -3.290121966 | 0.038319579 |
| HOXB5 | -3.3918095 | 0.037338204 |
| NAPSA | -3.395001808 | 0.000120894 |
| SNORD1C | -3.428306366 | 0.006470203 |
| HSD17B6 | -3.469566227 | 0.002020306 |
| SFTPA2 | -3.545110348 | 0.000286447 |
| MIR627 | -3.709579693 | 0.03565759 |
| SNORD89 | -3.770612917 | 9.34524E-05 |
| MIR502 | -3.925731937 | 0.028017148 |
| LRCH2 | -4.160563746 | 0.023991598 |
| C4BPA | -4.195167519 | 2.68345E-05 |
| OR10H3 | -4.419053963 | 0.010361621 |
| SFTPD | -4.657086513 | 1.13E-07 |
| MIR4699 | -5.010188905 | 0.042245081 |
| FOXD4L4 | -5.016512082 | 0.048127937 |
| SFTPC | -5.14648419 | 3.63835E-06 |
| MIR153-1 | -5.467676044 | 0.027061431 |
| MIR4454 | -6.157411023 | 0.048011675 |
| SPANXN3 | -6.354922931 | 0.018211349 |
| OR5R1 | -6.484916573 | 0.0275299 |

Table S4: Differentially expressed genes in isolated AEC2 cells. List of all differentially expressed genes in AEC2 cells of patients with IPF with history of moderate COVID compared to IPF alone group. *p < 0.05

| **Gene Symbol** | **log2FoldChange** | **p-value** |
| --- | --- | --- |
| KIR2DL5A | 5.397653808 | 0.016089474 |
| KIR2DL5B | 5.397653808 | 0.016089474 |
| VTRNA1-1 | 5.031492372 | 0.000365602 |
| HLA-DRA | 4.921522685 | 0.000597902 |
| SNORA80E | 4.717779059 | 0.000152454 |
| SNORD17 | 4.477612721 | 6.05026E-05 |
| SNORA23 | 3.431839093 | 0.026176277 |
| SNORA61 | 3.188243453 | 0.008675872 |
| LZTS1-AS1 | 2.587450725 | 0.014538294 |
| DNAH7 | 2.546540343 | 0.00905553 |
| FCGR2C | 2.469659051 | 0.02682566 |
| CD207 | 2.439678719 | 0.030261106 |
| TTC18 | 2.353983081 | 0.009894815 |
| ATP6AP1L | 2.312285443 | 0.047090208 |
| MTRNR2L1 | 2.288217748 | 0.038376829 |
| COL28A1 | 2.273379877 | 0.018497398 |
| ECT2L | 2.234736524 | 0.023024401 |
| DNAAF1 | 2.22729213 | 0.026321245 |
| HSPA7 | 2.222016146 | 0.034082165 |
| DMD | 2.216832401 | 0.020226362 |
| C9orf117 | 2.184725586 | 0.030185871 |
| SPEF2 | 2.175062909 | 0.019556264 |
| DNAH12 | 2.15967003 | 0.026783859 |
| TTC29 | 2.154888655 | 0.049403481 |
| CFAP43 | 2.151752631 | 0.024993656 |
| FCN1 | 2.115877066 | 0.017534869 |
| DNAH11 | 2.099955768 | 0.0121468 |
| CTSK | 2.092680294 | 0.032515675 |
| WDR16 | 2.082394809 | 0.023167619 |
| SPAG17 | 2.076150566 | 0.023433791 |
| ARMC4 | 2.07084594 | 0.041889062 |
| DTHD1 | 2.051065132 | 0.035280007 |
| DNAH6 | 2.036091703 | 0.023107851 |
| FAM179A | 2.021614276 | 0.021528449 |
| FLJ38122 | 2.020426808 | 0.034420453 |
| DNAH3 | 2.016736473 | 0.019140727 |
| DYDC2 | 1.992201656 | 0.033133807 |
| DYNC2H1 | 1.96709575 | 0.025191227 |
| LRRC48 | 1.965614165 | 0.032692264 |
| NEK10 | 1.962769032 | 0.04630508 |
| CCDC17 | 1.945371707 | 0.049543534 |
| DNAH10 | 1.940103977 | 0.015216936 |
| VPS37D | 1.937532981 | 0.049668664 |
| TFRC | 1.906542861 | 0.038284626 |
| NRAS | 1.896983097 | 0.046600566 |
| DNAH9 | 1.891777477 | 0.026752114 |
| CCR5 | 1.870380541 | 0.046907274 |
| FRMPD2 | 1.869601271 | 0.039655005 |
| LRRIQ1 | 1.856734813 | 0.047182072 |
| VWA3B | 1.842999101 | 0.025305093 |
| DLEC1 | 1.841309502 | 0.021849274 |
| GPR162 | 1.831540063 | 0.047330082 |
| EFHC1 | 1.830483025 | 0.043601325 |
| FYN | 1.819059581 | 0.041812059 |
| AK7 | 1.816090308 | 0.036846331 |
| MAATS1 | 1.809202748 | 0.038985349 |
| DNAI1 | 1.809172618 | 0.034302175 |
| PRR29 | 1.801115703 | 0.035552908 |
| TNFAIP8L1 | 1.795606497 | 0.039717386 |
| FANK1 | 1.792870404 | 0.037116261 |
| IL7R | 1.765667704 | 0.049862158 |
| CFAP44 | 1.764223612 | 0.04430515 |
| CDHR3 | 1.758209989 | 0.049039375 |
| TSNAXIP1 | 1.7465392 | 0.025669709 |
| CD300E | 1.712973361 | 0.049692702 |
| CCDC114 | 1.694722772 | 0.047558702 |
| LOC100652824 | 1.668667989 | 0.045633652 |
| HYDIN | 1.632577133 | 0.041147938 |
| CCDC13 | 1.629632047 | 0.034928142 |
| FHAD1 | 1.604124088 | 0.043729229 |
| PRUNE2 | 1.562621377 | 0.044411293 |
| WDR65 | 1.498272144 | 0.0488724 |
| C1R | -1.617968463 | 0.044944537 |
| S100A14 | -1.658675848 | 0.041553409 |
| DMBT1 | -1.735351765 | 0.019126685 |
| KCNJ15 | -1.73797921 | 0.016268025 |
| ALPL | -1.873416324 | 0.030688501 |
| MUC1 | -1.885521044 | 0.007908764 |
| MT2A | -1.906008515 | 0.049514835 |
| IFITM1 | -1.948853324 | 0.034816929 |
| MMP1 | -2.054840145 | 0.04454938 |
| TNFRSF10C | -2.058135055 | 0.033825713 |
| AQP1 | -2.067705271 | 0.02312465 |
| CSF3R | -2.148122732 | 0.009702317 |
| FMO2 | -2.202365054 | 0.039738211 |
| KRT13 | -2.27248481 | 0.015722446 |
| IFITM3 | -2.345000113 | 0.002588021 |
| NAPSA | -2.383971802 | 0.010238011 |
| C4BPA | -2.453915993 | 0.012764855 |
| KRT5 | -2.501423234 | 0.009529988 |
| IL1R2 | -2.578939879 | 0.01774989 |
| SFTPA2 | -2.582280403 | 0.009367131 |
| SFTPD | -2.610939539 | 0.009784061 |
| SCN5A | -2.628491352 | 0.031354728 |
| LOC646522 | -2.66816229 | 0.043280363 |
| ANXA8 | -2.736214753 | 0.017114933 |
| CNFN | -2.87771633 | 0.036468542 |
| CXCR1 | -2.923546353 | 0.015802048 |
| LINC01356 | -2.975351401 | 0.028243134 |
| DCD | -3.04381863 | 0.031553295 |
| PASD1 | -3.080565847 | 0.035358422 |
| KLK8 | -3.133221105 | 0.020739343 |
| IFITM2 | -3.162667332 | 0.000497445 |
| FTMT | -3.267665006 | 0.048449637 |
| DLEU1-AS1 | -3.296314428 | 0.028494929 |
| CXCR2 | -3.333352731 | 0.00195785 |
| LINC00470 | -3.391480966 | 0.019585477 |
| KRT6A | -3.405615659 | 0.000674322 |
| WDFY3-AS2 | -3.406619716 | 0.00875671 |
| SFTPC | -3.441083949 | 0.009060005 |
| MIR6856 | -3.518223513 | 0.045858375 |
| LOC101927354 | -3.636740135 | 0.042756079 |
| LINC00668 | -3.671022346 | 0.02116979 |
| KRTAP6-2 | -3.783844893 | 0.048523179 |
| MIR1249 | -3.832881234 | 0.033857814 |
| NTS | -3.91772668 | 0.014144099 |
| CCL27 | -4.241896535 | 0.021061136 |
| MIR325HG | -4.317248024 | 0.019248083 |
| LOC644919 | -4.473982454 | 0.009023895 |
| MIR3143 | -4.569989208 | 0.032102869 |
| LOC100506682 | -4.595865023 | 0.036840521 |
| MIR502 | -4.602813964 | 0.010280227 |
| OR7A17 | -4.633885947 | 0.042124988 |
| OSGEPL1-AS1 | -4.652632099 | 0.005762216 |
| MIR3158-1 | -4.821481284 | 0.005974288 |
| MIR3158-2 | -4.821481284 | 0.005974288 |
| KIR2DS4 | -4.93222171 | 0.022561756 |
| MIR326 | -5.000331229 | 0.006156496 |
| BGLT3 | -5.093854017 | 0.005895986 |
| MIR1253 | -5.320441912 | 0.034628396 |
| MIR1322 | -5.320441912 | 0.034628396 |
| RPL23AP32 | -5.400012337 | 0.003741536 |
| MIR145 | -5.530372515 | 0.01413267 |
| TGIF2LX | -5.721115671 | 0.016032937 |
| MIR15B | -5.926538958 | 0.00883066 |
| MIR1293 | -5.944236276 | 0.006035754 |
| KRTAP4-8 | -6.193951586 | 0.001626242 |
| MIR4699 | -6.370716892 | 0.001531473 |
| MIR4760 | -6.37816428 | 0.011825312 |
| BCYRN1 | -6.620570857 | 0.002456677 |
| MIR3938 | -6.722533279 | 0.005357435 |
| KIR3DL1 | -6.745592832 | 0.000132178 |
| CST11 | -6.749571572 | 0.001523546 |

Table S5: Gene ontology (GO) term enrichment of the top 20 differentially expressed genes in AEC2 cells of patients with IPF along with history of moderate COVID and COVID-ARDS, IPF alone groups.

| **Lung Disease Group** | **Gene ontology** |
| --- | --- |
| IPF with COVID history vs COVID-ARDS | \| Gobp_Positive_Regulation_Of_Type_2_Immune_Response.  Gobp_T_Helper_2_Cell_Differentiation.  Hp_Blurred_Vision.  Gobp_Positive_Regulation_Of_Interleukin_4_Production.  Gobp_Interleukin_4_Production.  Gobp_Regulation_Of_Type_2_Immune_Response.  Gobp_Positive_Regulation_Of_Cellular_Extravasation.  Gobp_Negative_Regulation_Of_Calcium_Ion_Transport_Into_Cytosol.  Hp_Lymphedema.  Gobp_Negative_Regulation_Of_Anoikis.  Gobp_Regulation_Of_Intrinsic_Apoptotic_Signaling_Pathway_In_  Response_To_Dna_Damage_By_P53_Class_Mediator.  Gobp_Negative_Regulation_Of_Intrinsic_Apoptotic_Signaling_  Pathway_By_P53_Class_Mediator.  Gobp_Endoplasmic_Reticulum_Calcium_Ion_Homeostasis.  Gobp_Somatic_Diversification_Of_Immunoglobulins.  Gobp_Positive_Regulation_Of_Mast_Cell_Activation.  Gobp_Regulation_Of_Response_To_Tumor_Cell.  Hp_Intellectual_Disability_Borderline.  Gobp_Positive_Regulation_Of_Transcription_From_Rna_  Polymerase_Ii_Promoter_Involved_In_Cellular_Response_To_  Chemical_Stimulus.  Gobp_Atp_Transport.  Gobp_Purine_Nucleotide_Transport. \| \| --- \| |
| IPF with COVID history vs IPF alone | Gomf_Microtubule_Motor_Activity.  Gomf_Atp_Dependent_Microtubule_Motor_Activity.  Gobp_Neural_Tube_Patterning.  Hp_Functional_Abnormality_Of_Male_Internal_Genitalia.  Hp_Decreased_Fertility_In_Males.  Hp_Abnormal_Male_Reproductive_System_Physiology.  Hp_Slurred_Speech.  Gobp_Microtubule_Depolymerization.  Gobp_Mitotic_Recombination.  Gobp_Negative_Regulation_Of_T_Cell_Mediated_Immunity.  Gomf_Tau_Protein_Kinase_Activity.  Gobp_Mature_B_Cell_Differentiation.  Hp_Retinal_Dystrophy.  Hp_Impaired_Vibratory_Sensation.  Gobp_Positive_Regulation_Of_Smoothened_Signaling_Pathway.  Hp_Infertility.  Gomf_Calcium_Release_Channel_Activity.  Gobp_Positive_Regulation_Of_Dna_Damage_Response_Signal_Transduction_By_P53_Class_Mediator.  Hp_Posterior_Subcapsular_Cataract.  Gobp_Dna_Dependent_Dna_Replication_Maintenance_Of_Fidelity. |
